# The oocyte cumulus complex regulates mouse sperm migration in the oviduct

**DOI:** 10.1038/s42003-022-04287-8

**Published:** 2022-12-03

**Authors:** Zhijuan Wang, Hongwei Wei, Zhanying Wu, Xiaodan Zhang, Yanli Sun, Longwei Gao, Wenqing Zhang, You-Qiang Su, Meijia Zhang

**Affiliations:** 1 Division of Cell, Developmental and Integrative Biology, School of Medicine, South China University of Technology, Guangzhou, 510006 P. R. China; 2 Shandong Provincial Key Laboratory of Animal Cells and Developmental Biology, School of Life Sciences, Shandong University, Qingdao, 266237 P. R. China

**Keywords:** Reproductive biology, Cell signalling

## Abstract

As the time of ovulation draws near, mouse spermatozoa move out of the isthmic reservoir, which is a prerequisite for fertilization. However, the molecular mechanism remains unclear. The present study revealed that mouse cumulus cells of oocytes–cumulus complexes (OCCs) expressed transforming growth factor-β ligand 1 (TGFB1), whereas ampullary epithelial cells expressed the TGF-β receptors, TGFBR1 and TGFBR2, and all were upregulated by luteinizing hormone (LH)/human chorionic gonadotropin (hCG). OCCs and TGFB1 increased natriuretic peptide type C (NPPC) expression in cultured ampullae via TGF-β signaling, and NPPC treatment promoted spermatozoa moving out of the isthmic reservoir of the preovulatory oviducts. Deletion of *Tgfb1* in cumulus cells and *Tgfbr2* in ampullary epithelial cells blocked OCC-induced NPPC expression and spermatozoa moving out of the isthmic reservoir, resulting in compromised fertilization and fertility. Oocyte-derived paracrine factors were required for promoting cumulus cell expression of TGFB1. Therefore, oocyte-dependent and cumulus cell-derived TGFB1 promotes the expression of NPPC in oviductal ampulla, which is critical for sperm migration in the oviduct and subsequent fertilization.

## Abstract (web summary)

Oocyte-dependent and cumulus cell-derived transforming growth factor-β promotes natriuretic peptide type C expression in mouse ampullary epithelial cells, which promotes sperm migration into the ampulla for fertilization.

## Introduction

In mammals, large numbers of spermatozoa are deposited in the female reproductive tract during coitus. A subpopulation of the ejaculated spermatozoa migrates to the lower isthmus of the oviduct and quickly binds to the epithelial cells to form a functional spermatozoa reservoir (known as the isthmic reservoir), where spermatozoa are sequestered for variable periods, hours to months, depending on the species^1^. In mice, spermatozoa are stored in the isthmic reservoir for several hours^2,3^. As the time of ovulation draws near, spermatozoa move out of the isthmic reservoir^4,5^. Finally, only a few spermatozoa reach the ampulla for fertilization^2^. The migration of spermatozoa from the isthmic reservoir to the ampulla is a prerequisite for fertilization and is believed to be achieved by one or a combination of factors, such as oviduct peristaltic contractions, adovarian flow of oviduct fluid^6^, cilia beating^7^, and/or signaling molecules in the oviductal microenvironment^8,9^.

During estrus, luteinizing hormone (LH) from the pituitary acts on the granulosa cells of preovulatory follicles to induce oocyte maturation and ovulation. Ovulated oocytes–cumulus complexes (OCCs) are picked up by the infundibulum and then are held in the ampulla for fertilization^10^. In vitro in cattle, the spermatozoa bound in the isthmic reservoir leave the reservoir as soon as the vital OCCs reach the ampulla^9^. In vivo in pig, the number of spermatozoa is increased in the ampulla and cranial isthmus segments after the transfer of OCCs into the oviductal ampulla^11^. The number of spermatozoa is dramatically lower in the oviductal lumen as the ovulated OCCs cannot enter the infundibulum of mice deficient in *miR-34b/c* and *miR-449a/b/c* (miR-dKO) without functional motile cilia^7^. Thus, the interaction between OCCs and the oviductal ampulla may initiate a signaling cascade that plays a crucial role in triggering spermatozoa to move out of the isthmic reservoir. OCC-derived factors could promote gene expression in the oviductal epithelial cells to regulate the physiological function of the oviduct^10,12^. We previously reported that OCCs induce the expression of natriuretic peptide type C (NPPC) in the oviductal ampulla^13^. NPPC can promote human and mouse spermatozoa motility by binding to its receptor natriuretic peptide receptor 2 (NPR2)^13,14^. Furthermore, spermatozoa derived from *Npr2* mutant mice are unable to reach the ampullae for fertilization^13^. Thus, OCC-induced NPPC in the ampulla may promote spermatozoa moving out of the isthmic reservoir for fertilization by increasing spermatozoa motility.

NPPC expression is upregulated by estrogen^15,16^, dexamethasone^17^, gonadotropin-releasing hormone^18^, and transforming growth factor-β (TGF-β)^19^ in various cell types. We recently reported that TGF-β in granulosa cells promotes NPPC expression to maintain oocyte meiotic arrest^20^. In mammals, the TGF-β family contains three highly conserved ligands, including TGFB1, TGFB2, and TGFB3^21^. These ligands bind to the type 2 receptor (TGFBR2) followed by the type 1 receptor (TGFBR1) to phosphorylate SMAD2 (Sma- and Mad-related protein 2), and SMAD3. Phosphorylated SMAD2 (p-SMAD2) and p-SMAD3 then form a complex with SMAD4 to regulate its target gene expression^22^. TGF-β ligands are expressed in cumulus cells^20,23^ and their receptors in the oviduct^24,25^. Therefore, we examined the role of TGF-β signaling in OCC-stimulated NPPC expression in the oviductal ampulla and the mechanisms of NPPC in promoting fertilization.

Based on this background, we hypothesize that oocyte-dependent and cumulus cell-derived TGFB1 promotes NPPC expression by binding to TGFBR in the ampullary epithelial cells. NPPC then promotes sperm migration from the isthmic reservoir to the ampulla for fertilization.

## Results

### LH/human chorionic gonadotropin (hCG) promotes TGFB1 expression in cumulus cells

We first detected the expression patterns of TGF-β ligands and receptors in OCCs and ampulla. In cumulus cells of OCCs, the relative abundance of *Tgfb1* mRNA was obviously higher than that of both *Tgfb2* and *Tgfb3* mRNAs (Supplementary Fig. 1a). *Tgfb2* was highly expressed in oocytes (Supplementary Fig. 1b, c), consistent with our previous findings^20^. TGFB1 mRNA and protein levels were significantly higher in cumulus cells than oocytes and ampulla (Supplementary Fig. 2). On the contrary, TGFBR1 and TGFBR2 mRNA and protein levels were significantly higher in ampulla than cumulus cells (Supplementary Fig. 2).

**Fig. 1:**
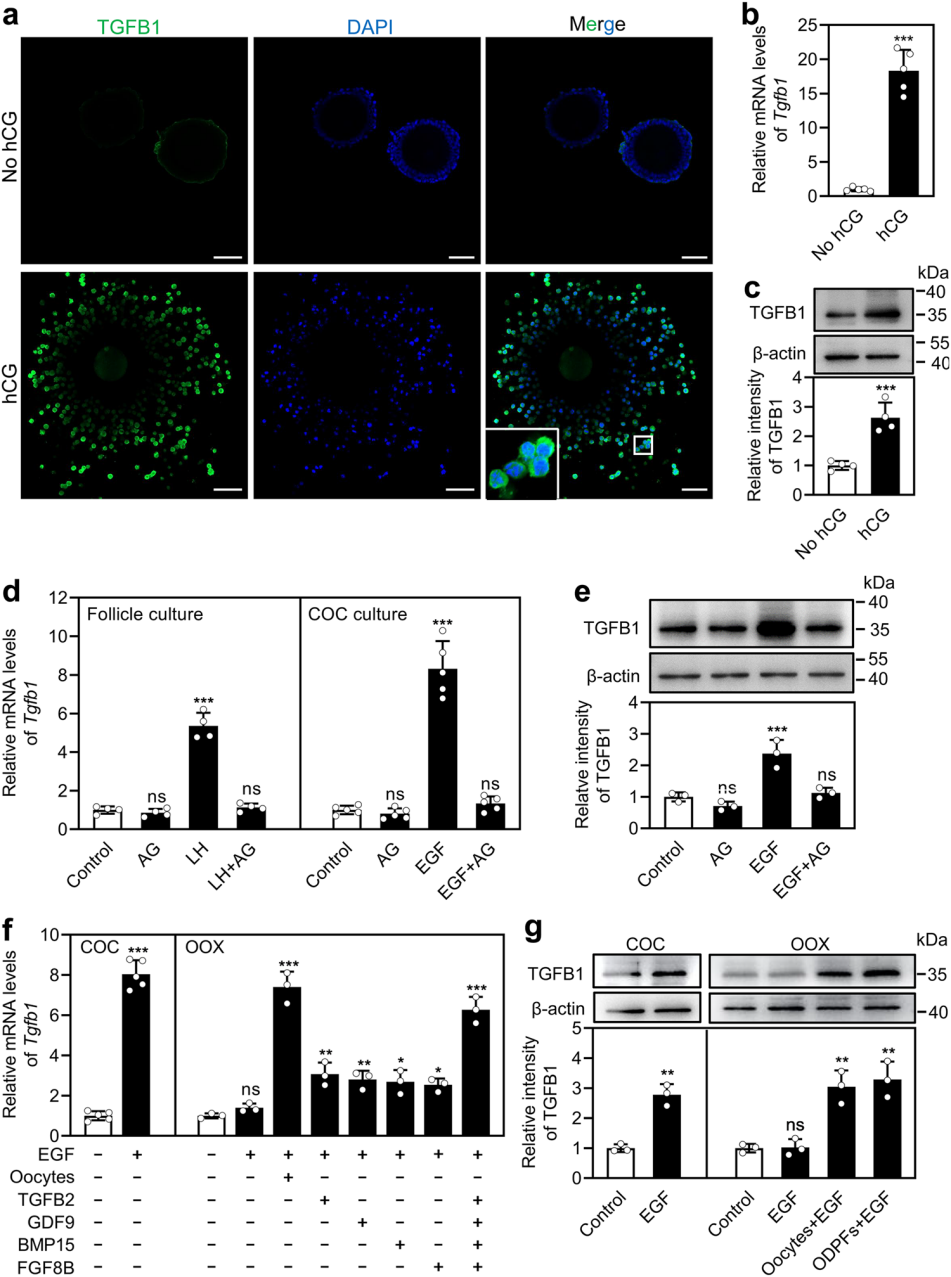
LH/hCG promotes TGFB1 expression in cumulus cells. Large follicles and COCs were isolated from eCG-primed mice. OOX cumulus cells were produced by microsurgically removing oocytes from the COCs. OCCs were collected from the ampullae of superovulated mice (at 13 h post-hCG treatment). **a** Immunofluorescence analysis of TGFB1 (green) in cumulus cells before and after hCG injection (at 13 h post-hCG treatment). (*n* = 3 independent experiments). Nuclei were counterstained by DAPI (blue). The small white box indicates the location of the enlarged area, as shown in the lower left corner. Scale bars represent 50 μm. **b**, **c** Comparison of steady-state mRNA (**b**) and protein (**c**) levels of TGFB1 in cumulus cells before and after hCG injection (at 13 h post-hCG treatment). (*n* = 5 independent experiments in (**b**) and *n* = 4 independent experiments in (**c**)). **d**, **e** The effects of LH and EGF on TGFB1 mRNA (**d**) and/or protein (**e**) expression in cumulus cells. Follicles were cultured in medium supplemented with LH (1 μg/ml) and/or AG1478 (1 μM) for 12 h, and COCs were cultured in medium supplemented with EGF (10 ng/ml) and/or AG1478 for 12 h. (*n* = 3–5 independent experiments). **f**, **g** The effects of EGF, oocytes, and ODPFs on TGFB1 mRNA (**f**) and protein (**g**) expression in cumulus cells. COCs or OOX cumulus cells were cultured in a medium supplemented with EGF, oocytes (3 oocytes/μl), GDF9 (500 ng/ml), BMP15 (500 ng/ml), FGF8B (FGF8, 100 ng/ml), and/or TGFB2 (50 ng/ml) for 12 h. ODPFs, GDF9 + BMP15 + FGF8B + TGFB2; AG, AG1478. (*n* = 3–5 independent experiments). β-actin was used as a loading control in (**c**), (**e**), and (**g**). Bars indicate the mean ± SD. Each data point represents a biologically independent experiment. Statistical analysis was performed by two-tailed unpaired Student’s *t*-test for two groups, and by one-way ANONA Tukey test for experiments involving more than two groups. ns, no significance (*P* ≥ 0.05). *P < 0.05, **P < 0.01, and ***P < 0.001.

The regulation of TGFB1 expression in cumulus cells was studied during LH/hCG-induced ovulation. Immunofluorescence analysis revealed that TGFB1 was located in the cytoplasm of cumulus cells (Fig. 1a). No green fluorescence signals were found in isotype-specific IgG staining, suggesting the specific binding of TGFB1 (Supplementary Fig. 3a). The fluorescence intensity of TGFB1 in the hCG treatment group was dramatically higher than that in the no hCG treatment group (Fig. 1a). In line with these findings, hCG treatment significantly increased TGFB1 mRNA and protein levels in cumulus cells (Fig. 1b, c). LH/hCG induces epidermal growth factor (EGF)-like growth factors in mural granulosa cells, which transactivates EGF receptor (EGFR) signaling in cumulus cells^26^. Thus, we studied the effect of EGFR signaling on LH/hCG-promoted TGFB1 expression. LH significantly increased *Tgfb1* mRNA levels in cumulus cells from cultured follicles (Fig. 1d), which was completely abolished by the EGFR tyrosine kinase inhibitor AG1478 (Fig. 1d). Furthermore, EGF significantly increased TGFB1 mRNA and protein levels in cumulus cells from cultured cumulus–oocyte complexes (COCs, Fig. 1d, e), which was also completely blocked by AG1478 (Fig. 1d, e). All these results suggest that LH–EGFR signaling promotes TGFB1 expression in cumulus cells.

EGFR signaling-induced gene expression in cumulus cells usually requires the participation of oocyte-derived paracrine factors (ODPFs)^27^. Therefore, we studied the possible role of ODPFs in regulating EGF-induced TGFB1 expression in cultured oocytectomized (OOX) complexes. EGF showed no effect on TGFB1 expression in OOX cumulus cells (Fig. 1f, g), but significantly increased TGFB1 mRNA and protein levels during coculture of OOX with fully-grown denuded oocytes, GDF9, BMP15, FGF8B, and/or TGFB2 (Fig. 1f, g). These results indicate that ODPFs participate in LH–EGFR signaling-promoted TGFB1 expression in cumulus cells.

### LH/hCG promotes TGFBR expression in the ampulla

**Fig. 2:**
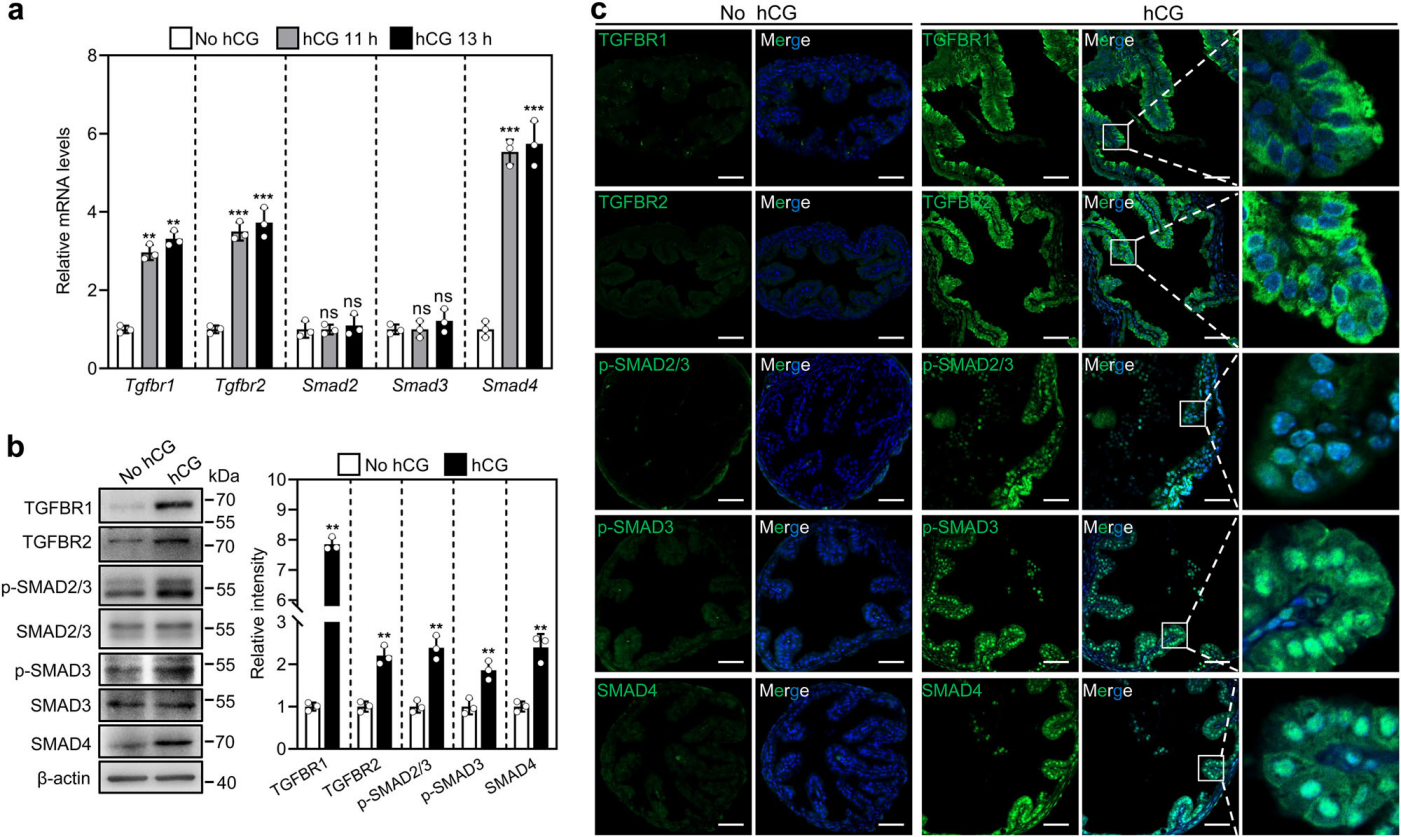
LH/hCG promotes TGFBR and SMAD4 expression in the ampulla. **a** Comparison of steady-state levels of *Tgfbr1*, *Tgfbr2*, *Smad2*, *Smad3*, and *Smad4* mRNA in the ampullae isolated from mice before and after hCG injection (*n* = 3 independent experiments). **b** Protein levels of TGFBR1, TGFBR2, SMAD4, p-SMAD2/3, and p-SMAD3 in the ampullae isolated from mice before and after hCG injection (at 13 h post-hCG treatment). (*n* = 3 independent experiments). Total levels of SMAD2/3 and SMAD3 were used as corresponding loading controls for p-SMAD2/3 and p-SMAD3, respectively, and β-actin was used as the loading control for TGFBR1, TGFBR2, and SMAD4. Bars indicate the mean ± SD. Each data point represents a biologically independent experiment. Statistical analysis was performed by one-way ANOVA Tukey test in (**a**) and by two-tailed unpaired Student’s *t*-test in (**b**). ns, no significance (*P* ≥ 0.05). **P < 0.01 and ***P < 0.001. **c** Immunofluorescence analysis of TGFBR1,TGFBR2, SMAD4, p-SMAD2/3, and p-SMAD3 (green) in the oviductal ampulla isolated from mice before and after hCG injection (at 13 h post-hCG treatment). (*n* = 3 independent experiments). Nuclei were counterstained by DAPI (blue). The amplified views of the boxed area are shown on the right-hand side. Scale bars represent 50 μm.

We studied the effect of LH/hCG on the expression of TGF-β receptors and their downstream signaling molecules in the ampulla. *Tgfbr1*, *Tgfbr2*, and *Smad4* mRNA levels were significantly increased in the ampulla at 11 h (without OCCs in the ampulla) and 13 h post-hCG (with OCCs in the ampulla) (Fig. 2a), suggesting that LH/hCG promotes the expression of these genes independent on OCC stimulation. The protein levels of TGFBR1, TGFBR2, SMAD4, p-SMAD2/3, and p-SMAD3 in the oviductal ampullae from hCG treatment group were significantly higher than those from no hCG treatment group (TGFBR1, *P* < 0.001; TGFBR2, *P* = 0.0012; SMAD4, *P* = 0.002; p-SMAD2/3, *P* = 0.0016; p-SMAD3, *P* = 0.0068. Figure 2b). Immunofluorescence analysis also showed that hCG treatment dramatically increased the cytoplasm accumulation of TGFBR1 and TGFBR2, and the nuclear accumulation of p-SMAD2/3, p-SMAD3, and SMAD4 (Fig. 2c). No fluorescence signals were found in isotype-specific IgG staining, suggest the specific binding of these primary antibodies (Supplementary Fig. 3b). Although hCG and LH bind to the same receptor, they may have different roles. We collected oviductal ampullae from mice at the diestrus and estrus stages to detect the effect of endogenous LH. The results showed that the gene and protein levels of TGFBR1, TGFBR2, and SMAD4 in the ampullae from estrous mice were significantly higher than those from diestrous mice (Supplementary Fig. 4). Thus, LH/hCG upregulates the expression of TGFBR and SMAD4 in the ampulla and activates TGF-β signaling pathway. In the ampulla, the relative abundance of *Lhcgr* (encoding the LH receptor) mRNA was very low (Supplementary Fig. 5a), and was lower than that of *Egfr* (encoding EGF receptor) mRNA (Supplementary Fig. 5b). Furthermore, EGF, but not LH, significantly increased the mRNA levels of *Tgfbr1*, *Tgfbr2*, and *Smad4* in cultured ampullae (Supplementary Fig. 5c, d). These results show that LH/hCG promotes the expression of TGFBR1, TGFBR2, and SMAD4 in the ampulla, possibly via the EGFR signaling pathway.

### OCC-stimulated NPPC in the ampulla promotes sperm migration in the oviduct

**Fig. 3:**
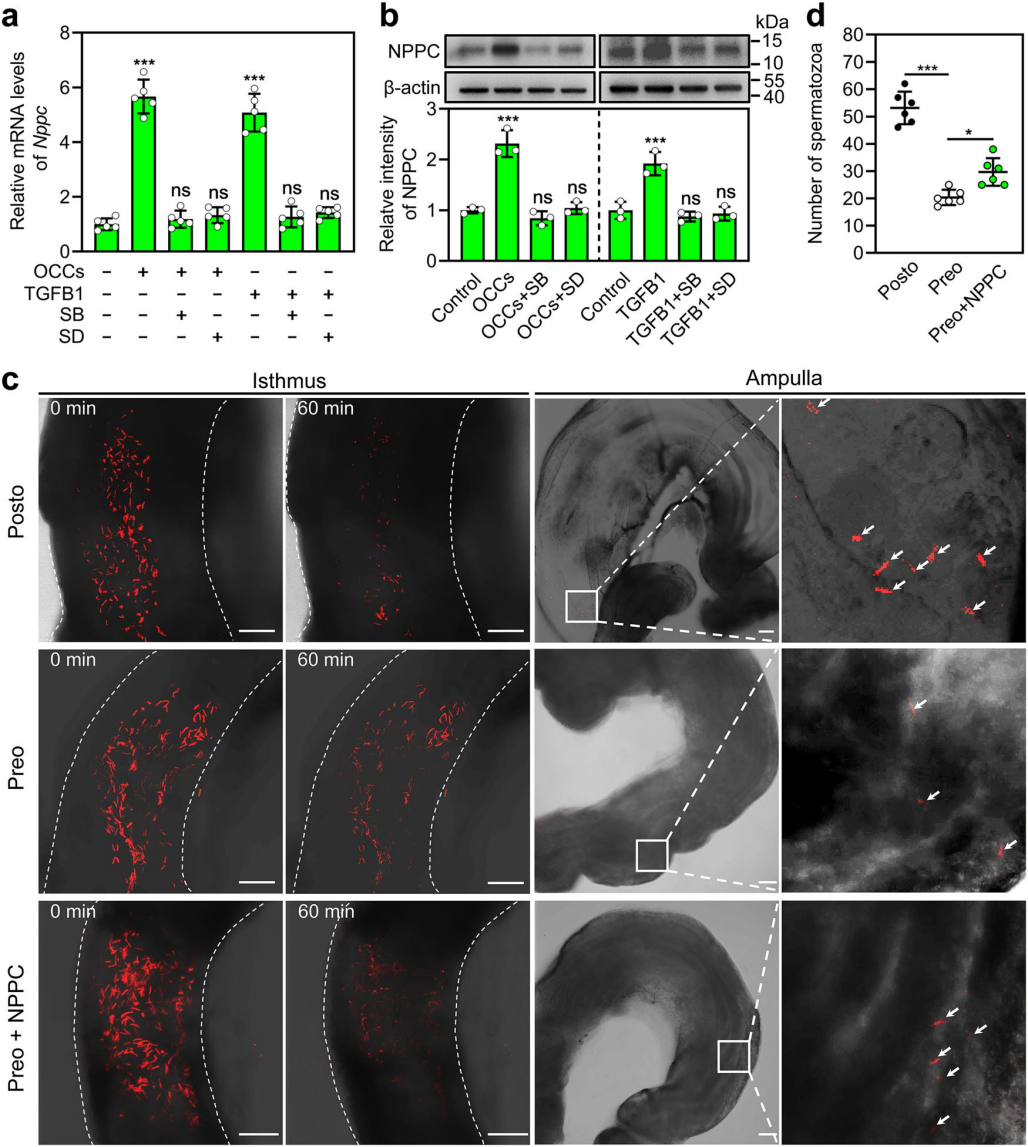
OCC-stimulated NPPC promotes sperm migration from the isthmic reservoir to the ampulla. **a**, **b** OCC and TGFB1-induced NPPC mRNA (**a**) and protein (**b**) levels were blocked by SB431542 and SD208. (*n* = 5 independent experiments in (**a**) and *n* = 3 independent experiments in (**b**). The ampullae were isolated from superovulated mice at 11 h post-hCG (without OCCs in the ampullae), and OCCs were collected from the ampullae of superovulated mice at 13 h post-hCG treatment. The ampullae were cultured with OCCs (3 OCCs/μl), TGFB1 (5 ng/ml), SB431542 (SB, 5 μM), and/or SD208 (SD, 1 μM) in a 50 μl drop for 3 h, and NPPC mRNA and protein levels were determined at the end of the culture. β-actin was used as a loading control. Each data point represents a biologically independent experiment. **c** Representative frames from the time-lapse imaging of the oviductal lower isthmus (left panel) and representative images of the ampulla at the end of the time-lapse imaging (right panel). (*n* = 6 mice in each group). The dashed line represents the edge of the lower isthmus. Oviducts were isolated from mice at the stated time. Amplified views of the boxed area are shown on the right-hand side. Arrow indicates spermatozoon in the oviductal ampullae. Scale bars represent 100 μm. Posto, postovulatory oviduct; Preo, preovulatory oviduct. **d** The number of spermatozoa in the ampullae at the end of the time-lapse imaging (*n* = 6 mice in each group). Each data point represents a single mouse. Statistical analysis was performed by one-way ANOVA Tukey test. Bars indicate the mean ± SD. ns, no significance (*P* ≥ 0.05). *P < 0.05 and ***P < 0.001.

Our previous work shows that ovulation induces *Nppc* expression in the oviductal ampullae^13^. OCCs promoted NPPC mRNA and protein expression in the cultured ampullae, which was completely blocked by the TGFBR1 kinase inhibitors, SB431542 and SD208 (Fig. 3a, b). We used TGFB1 to activate TGF-β signaling in the cultured ampullae since TGFB1 is highly expressed in the cumulus cells of OCCs. The addition of TGFB1 significantly increased the mRNA and protein levels of NPPC in the cultured ampullae (Fig. 3a, b), which was abolished by SB431542 and SD208 (Fig. 3a, b). Thus, OCCs stimulated NPPC expression in the ampulla by cumulus cell-derived TGFB1 binding to TGFBR.

We examined the role of OCC-stimulated NPPC in spermatozoa moving out of the isthmic reservoir by monitoring sperm migration in the lower isthmus using B6D2-Tg (CAG/su9-DsRed2, Acr3-EGFP) RBGS002Osb (RBGS) mice whose spermatozoa express a red fluorescent protein in their mitochondria^3^. Whole oviducts were dissected from female mice after coitus at a stated time, and the movement of spermatozoa in the lower isthmus was observed using a Zeiss LSM 880 confocal laser-scanning microscope. In postovulatory oviducts with OCC-stimulated NPPC expression, large numbers of spermatozoa moved out of the isthmic reservoir in the observed time, and many shifted in the flow of the adovarian oviduct fluid (Supplementary Video 1). A few spermatozoa were observed in the ampullae after time-lapse imaging (60 min) (Fig. 3c, d). In contrast, only a small amount of spermatozoa moved out of the isthmic reservoir in preovulatory oviducts without OCC-stimulated NPPC expression (Supplementary Video 1), and fewer spermatozoa were detected in the ampullae (Fig. 3c, d). The addition of NPPC into the preovulatory oviducts promoted the migration of spermatozoa from the isthmic reservoir to the oviductal lumen (Supplementary Video 1), and a few spermatozoa were detected in the ampullae (Fig. 3c, d). Taken together, these results show that OCC-stimulated NPPC promotes sperm migration from the isthmic reservoir to the ampulla.

### Conditional deletion of *Tgfb1* and *Tgfbr2* blocks NPPC expression and sperm migration in the oviduct

**Fig. 4:**
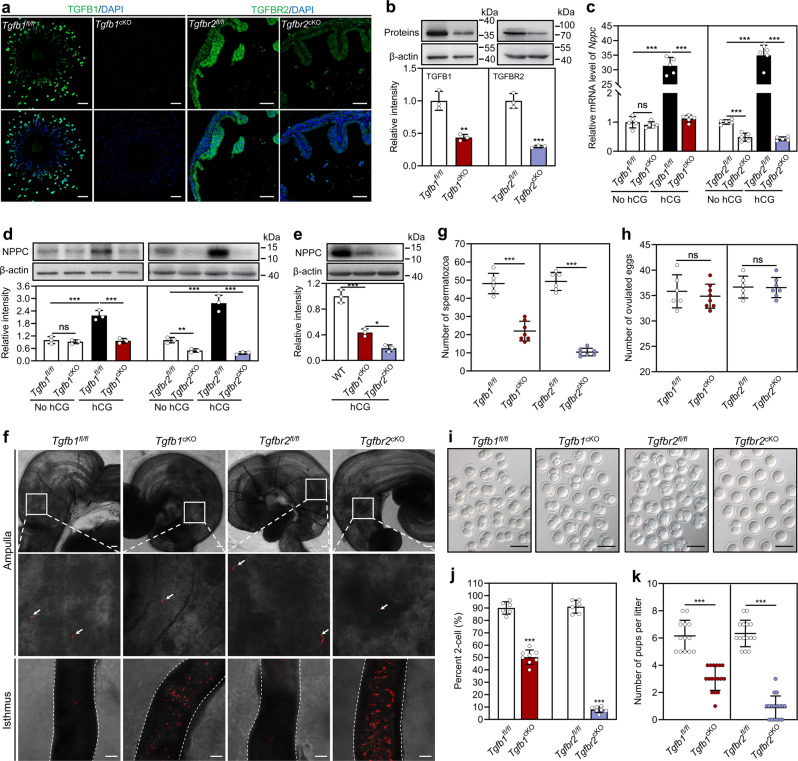
Conditional deletion of *Tgfb1* in cumulus cells and *Tgfbr2* in ampullary epithelial cells blocks NPPC expression and sperm migration in the oviduct. **a**, **b** Knockout efficiency of TGFB1 in cumulus cells and TGFBR2 in the oviductal ampullae were detected by immunofluorescence (**a**) and Western blotting (**b**). (*n* = 3 mice in each group). OCCs and ampullae were isolated from superovulated mice at 13 h post-hCG treatment. Nuclei were counterstained by DAPI (blue). Scale bars represent 50 μm. **c**, **d** NPPC mRNA (**c**) and protein (**d**) levels in the ampullae isolated from mice before and after hCG injection (at 13 h post-hCG treatment). (*n* = 5 independent experiments in (**c**) and *n* = 3 independent experiments in (**d**)). **e** NPPC protein levels in the ampullae isolated from superovulated mice at 13 h post-hCG treatment (*n* = 3 independent experiments). **f** Representative images of the oviductal ampulla and lower isthmus isolated from mice ~3 h postcopulation (at 16 h post-hCG treatment). (*n* = 6-7 mice in each group). The dashed line represents the edge of the oviductal lower isthmus. Amplified views of the boxed area are shown in the lower pane. Arrow indicates spermatozoon in the oviductal ampulla. Scale bars represent 100 μm. **g** The number of spermatozoa in the ampullae. Oviducts were isolated from mice ~3 h postcopulation (at 16 h post-hCG treatment), and the number of spermatozoa in the ampullae were counted for each mouse. (*n* = 6–7 mice in each group). **h** Number of oocytes ovulated by *Tgfb1*^*fl/fl*^, *Tgfb1*^cKO^, *Tgfbr2*^*fl/fl*^, and *Tgfbr2*^cKO^ mice (*n* = 6–8 mice in each group). **i** Representative images of the two-cell embryos. Scale bars represent 100 μm. **j**, **k** Rate of two-cell embryos (**j**) and the number of pups per litter (**k**). (*n* = 6–17 mice in each group). Two-cell embryos were obtained from mice at 1.5 days post coitum. β-actin was used as a loading control in (**b**, **d**, and **e**). Bars indicate the mean ± SD. Each data point represents a biologically independent experiment in (**b**–**e**), or represents a single mouse in (**g**, **h**, **j**, and **k**). Statistical analysis was performed by two-tailed unpaired Student’s *t*-test in (**b**, **g**, **h**, **j**, and **k**), and by one-way ANOVA Tukey test in (**c**–**e**). ns, no significance (*P* ≥ 0.05). *P < 0.05, **P < 0.01, and ***P < 0.001.

To study whether cumulus cell-derived TGFB1 promoted NPPC expression in the ampulla, we produced conditional knockout (cKO) mice with inactivated TGFB1 in the cumulus cells (*Tgfb1*^cKO^) by crossing *Tgfb1*^*fl/fl*^ mice with *Fshr*–*Cre* mice, and cKO mice with inactivated TGFBR2 in epithelial cells (*Tgfbr2*^cKO^) by crossing *Tgfbr2*^*fl/fl*^ mice with *Wnt7a*–*Cre* mice. Immunofluorescence analysis revealed that TGFB1 and TGFBR2 were specifically reduced in cumulus cells and ampullary epithelial cells, respectively (Fig. 4a). The knockout efficiency was also confirmed by Western blotting (Fig. 4b). Histological and co-staining analyses indicated that *Tgfbr2* deletion in epithelial cells had no obvious effect on the microstructure of the oviducts or distribution or the relative number of ciliated and secretory cells in the oviductal epithelium (Supplementary Fig. 6). We also stained sections of the oviduct with a marker of smooth muscle (αSMA), and found there were no differences in the thickness of myosalpinx in either the ampullary or isthmic regions between the *Tgfbr2*^*fl/fl*^ and *Tgfbr2*^cKO^ mice (Supplementary Fig. 7). hCG-induced ovulation-related NPPC mRNA and protein levels in the oviductal ampullae of *Tgfb1*^*fl/fl*^ and *Tgfbr2*^*fl/fl*^ mice (Fig. 4c, d), which was not detected in those of *Tgfb1*^cKO^ or *Tgfbr2*^cKO^ mice (Fig. 4c, d). In line with this, luciferase immunoassay analysis revealed that significantly lower concentrations of NPPC in the ampullae from *Tgfb1*^cKO^ and *Tgfbr2*^cKO^ mice compared with the corresponding control mice (Supplementary Fig. 8). Thus, cumulus cell-derived TGFB1 promoted ampullary NPPC expression via TGFBR. Interestingly, NPPC protein levels in the ampullae of *Tgfbr2*^cKO^ mice were significantly lower than those of *Tgfb1*^cKO^ mice in the hCG treatment group (Fig. 4e), and *Tgfbr2* deletion also decreased NPPC levels in the ampullae in the no hCG treatment group (Fig. 4c, d). This suggests that TGF-β ligands from the oviductal microenvironment promote NPPC expression (basal levels) in the ampulla by binding to TGFBR2, which is independent of ovulation.

We next examined the physiological role of TGFB1-promoted NPPC in sperm migration in the oviduct. In postovulatory oviducts of *Tgfb1*^*fl/fl*^ and *Tgfbr2*^*fl/fl*^ mice, large numbers of spermatozoa moved out of the isthmic reservoir, and many shifted in the flow of the adovarian oviduct fluid (Supplementary Video 2, 3). In contrast, small numbers of spermatozoa moved out of the isthmic reservoir in the postovulatory oviducts of *Tgfb1*^cKO^ and *Tgfbr2*^cKO^ mice (Supplementary Video 2, 3). The addition of NPPC to the postovulatory oviducts of *Tgfb1*^cKO^ and *Tgfbr2*^cKO^ mice obviously promoted spermatozoa moving out of the isthmic reservoir (Supplementary Video 2, 3). We also examined the distribution of spermatozoa in the oviducts from mice at ~3 h post-copulation (at 16 h post-hCG treatment). Compared with the corresponding control mice, fewer spermatozoa were observed in the ampullae, and many more spermatozoa in the isthmic reservoir of *Tgfb1*^cKO^ and *Tgfbr2*^cKO^ mice (Fig. 4f, g). There were no differences in the total number of spermatozoa in the oviducts between *Tgfbr2*^*fl/fl*^ and *Tgfbr2*^cKO^ mice (Supplementary Fig. 9), suggesting that conditional deletion of *Tgfbr2* in epithelial cells has no overt effect on spermatozoa entering the oviducts. Therefore, cumulus cell-derived TGFB1 induces NPPC expression in the ampulla, and then NPPC promotes sperm migration in the oviduct.

We further examined the effects of *Tgfb1* and *Tgfbr2* deletion on fertilization and found that 50.3% of oocytes in *Tgfb1*^cKO^ mice and 7.9% of oocytes in *Tgfbr2*^cKO^ mice underwent successful fertilization and development to the two-cell stage after natural mating (Fig. 4i, j). Fertility testing revealed that *Tgfb1*^cKO^ mice had significantly fewer pups per litter than *Tgfb1*^*fl/fl*^ mice (3.05 ± 0.90 versus 6.15 ± 1.14, *P* < 0.001; Fig. 4k). Unlike *Tgfbr2*^*fl/fl*^ mice that produced 6.3 pups per litter, *Tgfbr2*^cKO^ mice only produced 0.9 pups per litter (*P* < 0.001; Fig. 4k): six *Tgfbr2*^cKO^ mice with a copulatory plug failed to produce pups, and the other eleven mice with copulatory plug produced 1–3 pups (Fig. 4k). However, conditional deletion of *Tgfb1* and *Tgfbr2* had no effect on ovulation, and the ovulated oocytes could be fertilized in vitro and underwent the first round of cleavage normally (Fig. 4h, Supplementary Fig. 10). Collectively, *Tgfb1* deletion in cumulus cells and *Tgfbr2* deletion in epithelial cells blocked ovulation-induced NPPC expression in the ampulla, resulting in a dramatic decrease in sperm migration in the oviduct and compromised fertility.

### Conditional deletion of *Tgfbr2* in epithelial cells changes the oviduct transcriptome

**Fig. 5:**
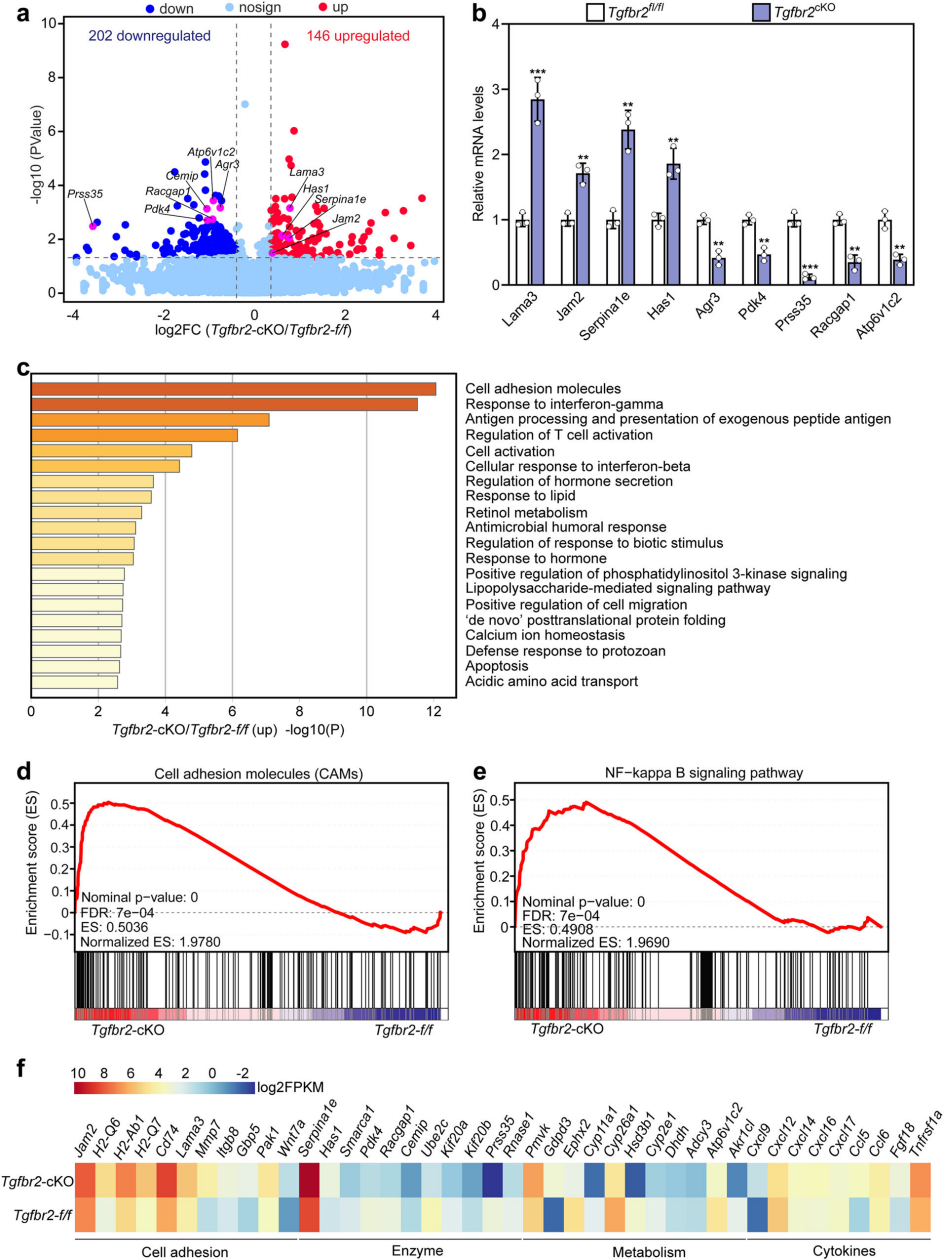
Distortion of the oviduct transcriptome in *Tgfbr2*^cKO^ oviduct. **a** Volcano plot illustrating the differential expression genes in the oviductal cells (at 13 h post-hCG) of *Tgfbr2*^*fl/fl*^ and *Tgfbr2*^cKO^ mice determined by RNA-seq analysis (*n* = 3 mice in each group). The pink spots denote the transcripts that were validated by qRT-PCR. **b** qRT-PCR validating changes in representative transcripts selected from RNA-seq data (*n* = 3 independent experiments). Bars indicate the mean ± SD. Each data point represents a single mouse. Statistical analysis was performed by a two-tailed unpaired Student’s *t*-test. **P < 0.01 and ***P < 0.001. **c** Bar graph showing the enriched GO/KEGG terms associated with the significantly upregulated transcripts in oviducts isolated from the *Tgfbr2*^cKO^ mice identified by RNA-seq, with the color indicating *P* value (*n* = 3 mice in each group). **d**, **e** GSEA plots illustrating enrichment of gene sets of cell adhesion molecules (**d**) and NF-kappa B signaling pathway (**e**) in oviductal cells of *Tgfbr2*^*fl/fl*^ and *Tgfbr2*^cKO^ mice (*n* = 3 mice in each group). **f** Heatmap showing differences between the *Tgfbr2*^*fl/fl*^ and *Tgfbr2*^cKO^ oviducts in the expression of a group of transcripts involved in various processes (*n* = 3 mice in each group).

To uncover the potential molecular mechanisms attributing to sperm migration defects, transcriptome analysis was performed on the oviductal cells of *Tgfbr2*^*fl/fl*^ and *Tgfbr2*^cKO^ mice (at 13 h post-hCG. Supplementary Fig. 11a). A total of 348 transcripts were identified to be significantly dysregulated in the oviductal cells of *Tgfbr2*^cKO^ mice, of which 202 transcripts were downregulated and 146 transcripts were upregulated (Fig. 5a, Supplementary Data 1). The significant dysregulation of the representative transcripts was validated by qRT-PCR (Fig. 5b). Gene enrichment analysis showed that the upregulated transcripts were mainly enriched for cell adhesion and immune responses (Fig. 5c). The downregulated transcripts were mainly enriched for mitotic cell cycle (Supplementary Fig. 11b). Gene set enrichment analysis (GSEA) suggested that cell adhesion and NF-kappa B signaling were activated (Fig. 5d, e) and the cell cycle and cholesterol metabolism were inactivated in the oviductal cells of *Tgfbr2*^cKO^ mice (Supplementary Fig. 11c, d). Further analysis of gene enrichment showed that the expression of *Has1* was upregulated, and the expression of *Cemip* and *Serpina1e* was downregulated in the oviductal cells of *Tgfbr2*^cKO^ mice (Fig. 5f).

## Discussion

Stored spermatozoa move out of the isthmic reservoir during ovulation. The present study indicated that cumulus cell-derived TGFB1 from ovulated OCCs promoted NPPC expression by binding to its receptors in the ampullary epithelial cells, and then NPPC triggered sperm migration from the isthmic reservoir to the ampulla for fertilization.

A small number of spermatozoa moved out of the isthmic reservoir of the preovulatory oviducts without OCC-stimulated NPPC expression (Supplementary Video 1), which was promoted by the addition of NPPC (Supplementary Video 1). Large numbers of spermatozoa moved out of the isthmic reservoir of the postovulatory oviducts with OCC-stimulated NPPC expression (Supplementary Video 1), which was blocked in *Tgfb1*^cKO^ and *Tgfbr2*^cKO^ mice (Supplementary Videos 2 and 3). Thus, NPPC produced by cumulus cell-derived TGFB1 in the ampulla promotes sperm migration from the isthmic reservoir to the ampulla. The ovulated OCCs are wrapped by the ampulla to form a relatively closed microenvironment from the ampulla to the isthmus, which would be beneficial for NPPC diffusion from the ampulla to the isthmus. There is some evidence that mouse spermatozoa detach from the epithelium before they move out of the isthmic reservoir^4^. NPPC increases the motility of mouse and human spermatozoa by binding to NPR2^13,14^, which may promote spermatozoa detachment from the epithelial cells by overcoming the adhesive forces. The lack of cumulus matrix impairs oocyte fertilization in bikunin-deficient female mice^28^, and spermatozoa from *Npr2* mutant mice could not arrive at the ampulla for fertilization^13^, possibly because of sperm migration failure. In humans, TGFB1 and TGFBR are also expressed in the granulosa cells^29^ and oviduct^30^, respectively, which may promote NPPC expression in the ampulla for sperm migration in the oviduct.

The decrease in the number of spermatozoa migrating from the isthmic reservoir to the ampulla led to reduced fertilization in *Tgfb1*^cKO^ and *Tgfbr2*^cKO^ mice. *Tgfbr2*^cKO^ mice had lower levels of NPPC in the oviductal ampulla than *Tgfb1*^cKO^ mice, suggesting that conditional deletion of *Tgfbr2* in oviductal epithelial cells not only blocks OCC-stimulated NPPC expression but also decreases basal levels of NPPC in the ampulla. TGF-β ligands from the oviductal microenvironment, particularly TGFB1^31^, may promote NPPC expression via TGFBR in the oviduct. Lower levels of NPPC in the oviducts of *Tgfbr2*^cKO^ mice resulted in fewer spermatozoa moving out of the isthmic reservoir and lower numbers of two-cell embryos and litter size. Thus, NPPC produced in the ampulla is critical for sperm migration in the oviduct for fertilization. Although ovulated OCCs were unable to induce NPPC expression in the ampullae of *Tgfb1*^cKO^ mice, a small number of spermatozoa still moved out of the isthmic reservoir, consistent with the findings of a previous study that reported a small number of swimming-free spermatozoa observed in the oviductal lumen in miR-dKO mice without OCCs in the ampulla^7^. This small number of spermatozoa migrate from the isthmic reservoir to the ampulla, possibly by the induction of the basal levels of NPPC in the oviduct.

Conditional deletion of *Tgfb1* in cumulus cells resulted in around a 50% fertilization rate. A previous study shows that the global deletion of *Tgfb1* has a slight effect on the fertilization rate^32^. This may be because the mice with global deletion of *Tgfb1* have around 50% fewer spontaneously ovulated oocytes than wild-type mice. *Nppc* mRNA is identified in the secretory cells of ampullary epithelial cells^33^. In the present study, the deletion of *Tgfbr2* in the epithelial cells using *Wnt7a–Cre* blocked NPPC expression in the oviducts, resulting in a dramatic decrease in sperm migration in the oviduct and severely compromised fertilization, but had no effect on the oviductal microstructure or the ability to produce pups. The deletion of *Tgfbr1* and *Tgfbr2* in the smooth muscle compartment using *Amhr2*–*Cre* reportedly impairs the integrity and function of the oviduct and uterus but has no effect on fertilization^24,25^. This indicates that TGFBR signaling plays different roles in the oviductal epithelial cells and the smooth muscle compartment. This concept is supported by a previous study that reported that the functions of estrogen receptor α signaling are different in the oviductal epithelial cells and the smooth muscle compartment^34^.

The activation of the LH receptor by LH/hCG promoted TGFB1 expression in cumulus cells, consistent with a previous report that the mRNA and protein levels of TGFB1 are increased in cumulus cells when the follicles are cultured in the presence of LH^23^. EGF-like growth factors are mediators of LH/hCG action in cumulus cells^26^. Consistent with this, LH/hCG promoted TGFB1 expression in cumulus cells via the transactivation of EGFR. LH*–*EGFR signaling-induced TGFB1 expression in cumulus cells required the cooperation of ODPFs, similar to the regulation of cumulus expansion-related transcripts^27^. Thus, oocytes are involved in sperm migration by promoting TGFB1 expression in cumulus cells and then NPPC expression in the oviductal ampulla. The previous reports show that oocytes promote follicular development by controlling the proliferation and metabolic activities of granulosa cells^35^, and promote ovulation by participating in cumulus expansion^36^. Therefore, oocytes coordinate follicular development, ovulation, and fertilization. LH/hCG also promoted TGFBR expression in the ampulla, possibly via the EGFR signaling pathway. Thus, LH/hCG promotes the expression of TGFB1 in cumulus cells and TGFBR in the ampulla, which is a prerequisite for ovulation-stimulated NPPC expression and subsequent sperm migration in the oviduct.

The entire mount oviduct was used for transcriptomic analysis since there is a potential interaction between the stromal cells and epithelial cells^25^. In *Tgfbr2*^cKO^ mice, the upregulation of *Has1* and the downregulation of *Cemip* may prevent sperm migration in the oviduct by increasing hyaluronan levels^37^, and the upregulation of *Serpina1e* may impair the spermatozoa motility by inhibiting serine proteases^38^. The activation of cell adhesion and NF-kappa B signaling in the oviductal cells of *Tgfbr2*^cKO^ mice may impair spermatozoa moving out of the isthmic reservoir^39^. The inactivation of cholesterol metabolism in the oviductal cells of *Tgfbr2*^cKO^ mice may impair the production of steroid hormones, and then impair spermatozoa survival and release^8,40^. Thus, these changed transcripts in the oviduct of *Tgfbr2*^cKO^ mice may also participate in the block of sperm migration in the oviduct.

**Fig. 6:**
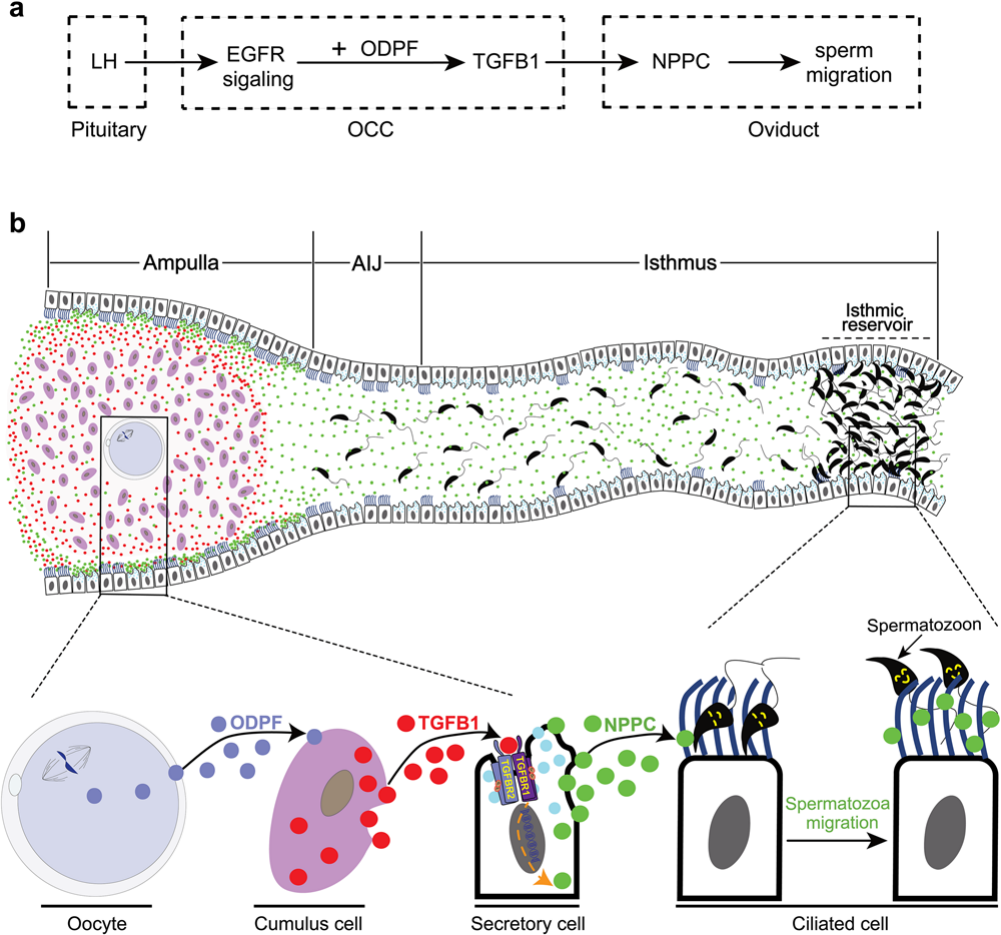
Schematic showing that cumulus cell-derived TGFB1 promotes NPPC expression in the ampulla for mouse sperm migration in the oviduct. **a**, **b** Molecular signaling (**a**) and cellular interaction (**b**) for sperm migration in the oviduct. When ovulation occurs, LH activates EGFR signaling in cumulus cells by EGF-like growth factors, and then cooperates with oocyte-derived paracrine factors (ODPF) to promote cumulus cell TGFB1 expression. TGFB1 induces NPPC production by binding to TGFBRs in the ampullary epithelial cells, and then promotes sperm migration from the isthmic reservoir to the ampulla for fertilization. AIJ, ampullary–isthmic junction.

The finding of the present study reveals a complex regulatory network involving ovulation-triggered mouse sperm migration in the oviduct. LH promotes the expression of TGFB1 in cumulus cells and TGFBR in the ampullary epithelial cells during ovulation. The interaction between OCCs and ampulla promotes NPPC expression by binding cumulus cell-derived TGFB1 to TGFBR in the ampullary epithelial cells. NPPC promotes sperm migration from the isthmic reservoir to the ampulla for fertilization. Oocytes are involved in sperm migration by promoting TGFB1 expression in cumulus cells (Fig. 6). The mechanism of ovulation-triggering sperm migration in the oviduct revealed here will be crucial for deciphering the potential role of NPPC in sperm migration in the oviduct in humans and other species.

## Methods

### Animals and chemicals

Female C57BL/6 J mice were purchased from Guangdong Medical Laboratory Animal Center. *Tgfb1*^*fl/fl*^, *Tgfbr2*^*fl/fl*^, and *Wnt7a*–*Cre* mice were purchased from The Jackson Laboratory (Bar Harbor, ME, USA). Mice with *Tgfb1* deletions in the cumulus cells were generated by crossing *Tgfb1*^*fl/fl*^ mice with *Fshr*–*Cre* mice^[20]^. Mice with *Tgfbr2* deletions in the epithelial cells were generated by crossing *Tgfbr2*^*fl/fl*^ mice with *Wnt7a*–*Cre* mice^34,41^. RBGS mice using for monitoring spermatozoa movement^3^ were purchased from RIKEN BioResource Center (Ibaraki, Tsukuba, Japan). Female mice (21–23 days old) were intraperitoneally injected with 5 IU equine chorionic gonadotropin (eCG) 48 h before use to stimulate follicle growth. In some experiments, female mice were intraperitoneally injected with 5 IU eCG followed by 5 IU hCG 48 h later to induce ovulation (superovulation). Fertility was analyzed in 6-week-old female mice. All mice were raised under controlled temperatures of 23 °C ± 2 °C with a 12/12 h light/dark cycle. Mouse experimental procedures and protocols were approved by the Institutional Animal Care and Use Committee of the South China University of Technology and were conducted in accordance with the institutional guides for the care and use of laboratory animals. Reagents were purchased from Sigma-Aldrich (St. Louis, MO, USA) unless otherwise stated.

### Isolation and culture of follicles, COCs, OOX cumulus cells, and oviductal ampulla

Large antral follicles (350–400 μm) and COCs were isolated from eCG-primed mice. OOX cumulus cells were produced by the microsurgical extirpation of the oocytes, but not the zona pellucida, from the COCs^42^. Follicles were cultured in a medium, supplemented with LH (1 μg/ml, human) and/or AG1478 (1 μM, EGF receptor inhibitor) on Millicell culture inserts (Millipore, Billerica, MA, USA). Groups of 40–50 COCs or OOXs were cultured in a medium, supplemented with EGF (10 ng/ml), AG1478, GDF9 (500 ng/ml), BMP15 (500 ng/ml), FGF8B (FGF8, 100 ng/ml), TGFB2 (50 ng/ml), and/or oocytes (3 oocytes/μl) in a 50 μl drop. Cumulus cells were collected after 12 h of culture to analyze TGFB1 mRNA and protein expression. Oviductal ampullae were collected from preovulatory mice (at 11 h post-hCG treatment) or eCG-primed mice using a pair of 26 gauge needles under a stereomicroscope^13^. Four oviductal ampullae were cultured in the medium, supplemented with 100 OCCs (25 OCCs/ampulla), TGFB1 (5 ng/ml), SB431542 (TGFBR1 kinase inhibitors, 5 μM), SD208 (TGFBR1 kinase inhibitors, 1 μM), LH, and/or EGF in a 50 μl drop. The ampullae were collected at the end of the culture to analyze mRNA and protein expression. In some experiments, ampullae were collected from the adult female mice at the estrus and diestrus stages for mRNA and protein analysis. The culture medium was bicarbonate-buffered minimum essential medium-alpha with Earle balanced salts (Thermo Fisher Scientific, Waltham, MA, USA), supplemented with 100 UI/ml penicillin–streptomycin, 0.23 mM pyruvate, and 3 mg/ml bovine serum albumin. For follicle culture, 1% insulin*–*transferrin*–*selenium (ITS, 13146) was added into the culture medium^43^. Cultures were carried out at 37 °C in an atmosphere of 5% CO_2_.

### Immunofluorescence and histology analyses

COCs from eCG-primed mice and OCCs from superovulated mice (at 13 h post-hCG treatment) were fixed in 4% paraformaldehyde (PFA) for 30 min at room temperature. After permeabilization with phosphate-buffered saline (PBS) containing 0.5% Triton X-100 for 5 min, samples were blocked with 5% bovine serum albumin for 1 h at room temperature. Oviducts were dissected from female mice under a dissection microscope before and after hCG injection (at 13 h post-hCG treatment), and fixed in 4% PFA at 4 °C overnight. After dehydration, the oviducts were embedded in paraffin and cut into 5-μm sections. Sections were blocked with 10% normal donkey serum. After blocking, all the samples were incubated with primary antibodies (Supplementary Table 1) and then incubated with Alexa Fluor 488 or 555-conjugated secondary antibodies (1:200, Thermo Fisher Scientific). Finally, samples were counterstained with DAPI. The isotype-specific immunoglobulins (IgG) at the same protein concentration as the primary antibodies were used for the negative control (Abcam, ab172730, Cambridge). Immunofluorescent staining was examined using a Zeiss LSM 800 confocal microscope (Zeiss, Oberkochen, Germany). Histological analysis was performed using sections from oviductal tissues stained with hematoxylin and eosin.

### Western blotting

In each group, 4–8 oviductal ampullae, 100–300 oocytes, or the cumulus cells from 50–100 OCCs or OOX were used. Total proteins were extracted in WIP buffer (Cell Chip Biotechnology, Beijing, China) with 1 mM phenylmethylsulfonyl fluoride on ice. Protein concentrations were quantified using the BCA Protein Assay Kit (Beyotime, Shanghai, China). Normalized protein amounts were separated by sodium dodecyl sulfate-polyacrylamide gel electrophoresis and then electrically transferred to polyvinylidene fluoride membranes (Millipore). After blocking with 5% nonfat milk in Tris-buffered saline (TBS) for 1 h at room temperature, the membranes were incubated with primary antibodies (Supplementary Table 1) overnight at 4 °C. Membranes were then washed three times for 5 min each in TBS with 0.1% Tween-20 and incubated with horseradish peroxidase-conjugated secondary antibodies (each diluted 1:5000, ZSGB-BIO, Beijing, China). Proteins were detected using SuperSignal West Pico Kit (Thermo Fisher Scientific) and visualized using the Tanon 5200 chemiluminescent imaging system (Tanon, Shanghai, China). β-actin was used as a loading control. Uncropped scans of Western blotting membranes are shown in Supplementary Fig. 12.

### RNA extraction and qRT-PCR analysis

In each group, 4 oviductal ampullae, 50 oocytes, or the cumulus cells from 40 OCCs, OOXs, or COCs were used. Total RNA was extracted and purified using the RNeasy micro-RNA Isolation Kit (Qiagen, Valencia, CA, USA), and no more than 1 μg RNA was reverse transcribed into cDNA using the QuantiTect Reverse Transcription System (Qiagen) according to the manufacturer’s instructions. qRT-PCR was conducted in 15-μl reaction volumes and analyzed on a Light Cycler 96 instrument (Roche, Basel, Switzerland) using a standard protocol. The reagent used for qRT-PCR is TransStart Tip Green qPCR SuperMix (TransGen, Beijing, China). Relative gene expression was quantified using the threshold cycle value and normalized using ribosomal protein L19 (*Rpl19*) as a housekeeping gene. Details of the qRT-PCR primers are shown in Supplementary Table 2.

### RNA-seq analysis

RNA-seq analysis was performed on triplicate samples of *Tgfbr2*^*fl/fl*^ and *Tgfbr2*^cKO^ oviducts (at 13 h post-hCG). Total RNA was extracted and purified from the oviducts using the RNeasy micro-RNA Isolation Kit (Qiagen) according to the manufacturer’s instructions. RNA quality was assessed on an Agilent 2100 Bioanalyzer (Agilent Technologies, Palo Alto, CA, USA) and checked using RNase-free agarose gel electrophoresis. Libraries were prepared with NEBNext Ultra RNA Library Prep Kit for Illumina (NEB #7530, New England Biolabs, Ipswich, MA, USA). The resulting cDNA library was sequenced using Illumina Novaseq6000 by Gene Denovo Biotechnology Co (Guangzhou, China). Correlation analysis was performed by R. Correlation of two parallel experiments provides the evaluation of the reliability of experimental results as well as operational stability. The differentially expressed transcripts were calculated by DESeq2 software between two different groups. Gene enrichment analysis (Gene Ontology/KEGG) was conducted using Metascape (http://metascape.org), a gene annotation and analysis resource, following the online instruction provided by the web developer. Gene set enrichment analysis (GSEA) was performed using the GSEA software.

### Measurement of NPPC levels

Oviductal ampullae were collected from the superovulated mice at 13 h post-hCG treatment. Ampullae from 10 mice per group were transferred into a 1.5-ml centrifuge tube for NPPC analysis. Samples were prepared as previously reported with slight modifications^13,44^. Briefly, ampullae were boiled in 1.0 M acetic acid for 5 min and then lysed using an ultrasonic cell disruptor (Scientz-IID, Ningbo, China). The samples were centrifuged at 20,000×*g* at 4 °C for 30 min, and the supernatant containing 1.0–5.0 mg of protein was lyophilized and assayed using fluorescent enzyme immunoassay kits (Phoenix Pharmaceuticals, Belmont, CA, USA) according to the manufacturer’s instructions.

### Analysis of ovulation, fertilization, and fertility

Ovulation was analyzed in female mice (21–23 days old) of different genotypes that were injected intraperitoneally with 5 IU eCG followed by 5 IU hCG 48 h later to induce ovulation. Oocytes collected from the oviductal ampulla of mice at 13 h post-hCG treatment were fertilized with capacitated epididymal spermatozoa isolated from cauda epididymis of 10-week-old fertile male mice. In vitro fertilization was performed in a human tubal fluid (HTF, MR-070) medium. After culturing at 37 °C in an atmosphere of 5% CO_2_ for 6 h, oocytes were washed, and fertilization was determined by the presence of two pronuclei at 8–9 h after IVF. The fertilized oocytes were then transferred to a K^+^ simplex optimized medium (KSOM) for further development^45^. Images of two-cell embryos were obtained using a Zeiss Axio Vert. A1 microscope. Fertility was analyzed by mating the 6-week-old female mice with 10-week-old fertile male mice. The number of pups per litter was recorded at birth.

### Analysis of sperm migration in the oviducts

Sperm migration was analyzed using RBGS male mice as their spermatozoa appear red under a fluorescent microscope. The 6-week-old superovulated female mice were caged with 10-week-old RBGS male mice starting at 9 h post-hCG injection. The presence of a copulation plug was checked every 20 min. Female mice mated at 10–11 h post-hCG (without OCCs in the ampulla) were used for preovulatory studies, and the female mice mated at 12–13 h post-hCG (with OCCs in the ampulla) were used for postovulatory studies. The ovary–oviduct–uterus complex was removed from the mouse ~30 min after copulation and placed in PBS. Entire oviducts were dissected from the ovary by opening the ovarian bursa. The oviduct was uncoiled and separated from the uterine horn by sharp dissection to yield single intact oviduct preparation comprising the infundibulum, ampulla, isthmus, and intramural segments. The mesosalpinx was carefully cut under a stereo microscope (Zeiss) to avoid pulling and damaging the tubes. All operations were performed in PBS. In some experiments, the oviducts were incubated with NPPC (10 nM) for ~30 min. The whole mount oviduct was transferred into a glass-bottomed dish (Nest, Jiangsu, China) and fixed to the bottom of the dish using low-melting agarose. The dish was then filled with PBS and maintained at a physiologically relevant temperature of 37 °C ± 0.5 °C in a thermal chamber. The movement of the spermatozoa within the lower isthmus was imaged using a Zeiss LSM 880 confocal microscope. Sites of the movies in the oviducts from preovulatory and postovulatory mice are shown in Supplementary Fig. 13. Time-lapse imaging was performed as previously described^46^ for 1 h with a time interval of ~1 min. Imaging files were processed using ImageJ software. At the end of the time-lapse imaging, the images of the oviductal ampullae were captured using a Zeiss LSM 880 confocal laser-scanning microscope. Then, spermatozoa in the ampullae were counted as previously described^34^. In some experiments, whole oviducts were dissected from female mice ~3 h postcopulation (at 16 h post-hCG treatment) to observe spermatozoa distribution and to count the total number of spermatozoa in the oviducts.

### Statistics and reproducibility

Statistical analyses were performed using GraphPad Prism version 8.3 (GraphPad Software, La Jolla, CA, USA). Two-tailed unpaired Student’s *t-*test was used for the analysis of the two groups, and *P* < 0.05 was considered significant. For experiments involving more than two groups, differences between groups were compared by one-way ANOVA Tukey test, and *P* < 0.05 was assigned to significantly different. Data are represented as the mean ± SD. All experiments were repeated at least three times using different mice, and detailed information was presented in the figure legends.

### Reporting summary

Further information on research design is available in the Nature Portfolio Reporting Summary linked to this article

## Supplementary information

Supplementary information

Description of Additional Supplementary Files

Supplementary Data 1

Supplementary Data 2

Supplementary Vedio 1

Supplementary Video 2

Supplementary Video 3

Reporting summary

## Data Availability

All data needed to evaluate the conclusions in this study are present in the manuscript and/or Supplementary information, and available from the corresponding author upon reasonable request. The source data underlying graphs presented in the main figures are provided in Supplementary Data 2. Uncropped scans of Western blotting results are provided in Supplementary Fig. 12. RNA-seq data are deposited in the NCBI Sequence Read Archive (accession number PRJNA888862).
