# Supplementary material for: The oocyte cumulus complex regulates mouse sperm migration in the oviduct
Source: Commun Biol. 2022 Dec 3;5:1327. doi: 10.1038/s42003-022-04287-8 (PMC9719508; doi:10.1038/s42003-022-04287-8)
Supplement: Supplementary file 1 — Supplementary information [file 42003_2022_4287_MOESM1_ESM.pdf]

Supplementary information

The oocyte cumulus complex regulates mouse sperm migration in the oviduct

Zhijuan Wang[1], Hongwei Wei[1], Zhanying Wu[1], Xiaodan Zhang[1], Yanli Sun[1], Longwei Gao[1], Wenqing Zhang[1], You-Qiang Su[2] and Meijia Zhang[1,*]

[1]Division of Cell, Developmental and Integrative Biology, School of Medicine, South China University of Technology, Guangzhou 510006, P. R. China.

[2]Shandong Provincial Key Laboratory of Animal Cells and Developmental Biology, School of Life Sciences, Shandong University, Qingdao 266237, P.R. China.

[*]To whom correspondence may be addressed. Email: zhangmeijia@scut.edu.cn.

This PDF file includes:

- Supplementary Figure and Figure legends
- Supplementary Tables (Table 1, 2)

Supplementary Figures and Figure legends

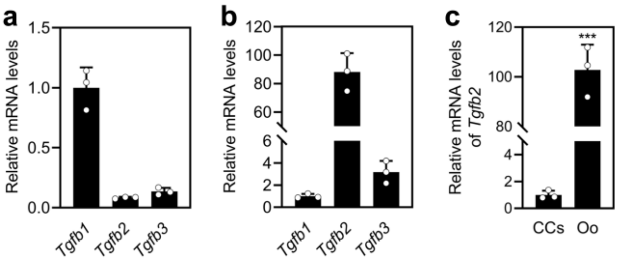

Supplementary Fig. 1 Expression patterns of TGF-β ligands in cumulus cells and oocytes after ovulation. OCCs were collected from superovulated mice at 13 h post-hCG treatment. **a**, **b** The mRNA levels of *Tgfb1*, *Tgfb2*, and *Tgfb3* in cumulus cells (**a**) and oocytes (**b**) from OCCs (n = 3 independent experiments). **c** Comparison of steady-state levels of *Tgfb2* mRNA in cumulus cells and oocytes (n = 3 independent experiments). Bars indicate the mean ± SD. Each data point represents a biologically independent experiment. Statistical analysis was performed by two-tailed unpaired Student's t-test. ***$P < 0.001$. CCs, cumulus cells; Oo, oocytes.

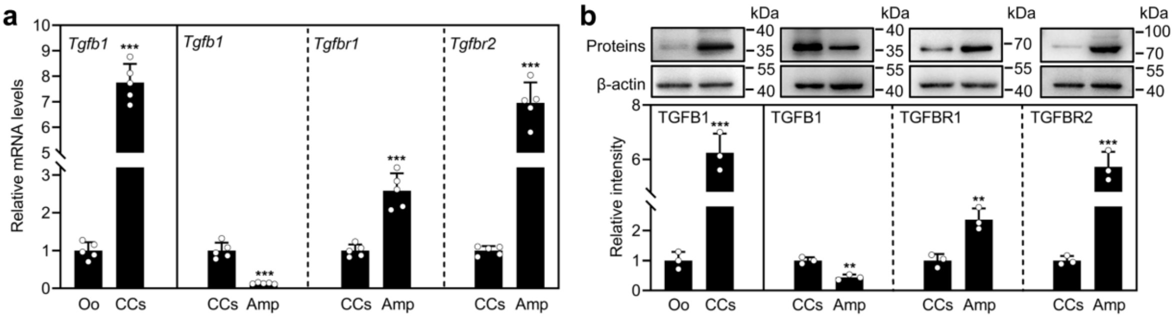

Supplementary Fig. 2 The expression patterns of TGFB1 and its receptors in cumulus cells and ampulla after ovulation. **a**, **b** Comparison of steady-state mRNA (**a**) and protein (**b**) levels of TGFB1, TGFBR1, and TGFBR2 in the oocytes, cumulus cells, and ampullae isolated from superovulated mice at 13 h post-hCG treatment. (n = 5 independent experiments in **a**, and n = 3 independent experiments in **b**). Oo, oocytes. CCs, cumulus cells. Amp, ampullae. The blot is the representative of three independent experiments. Bars indicate the mean ± SD. Each data point represents a biologically independent experiment. Statistical analysis was performed by two-tailed unpaired Student's t-test. **$P < 0.01$ and ***$P < 0.001$.

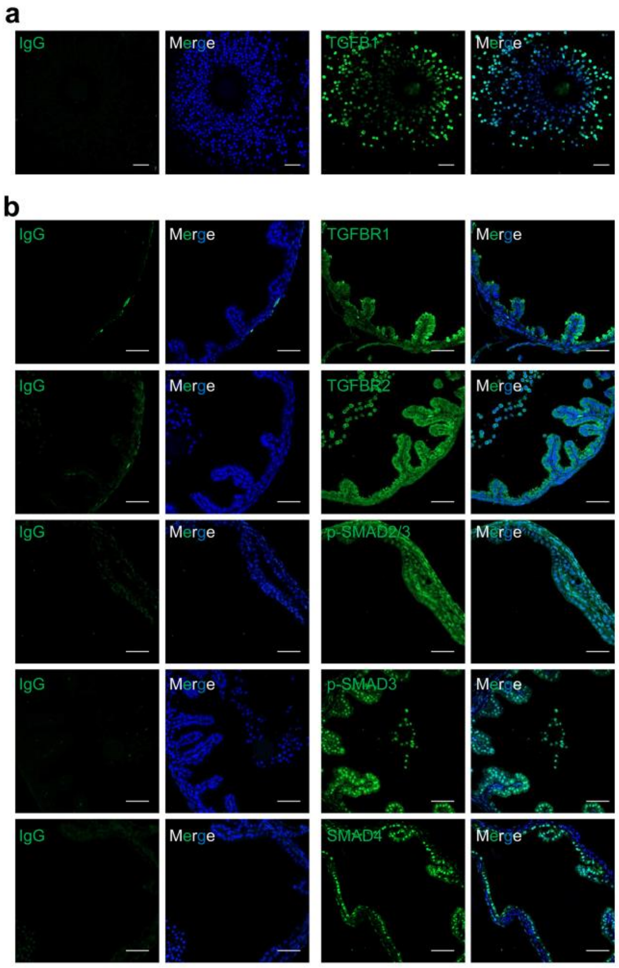

Supplementary Fig. 3 The specific binding of the primary antibodies. **a**, **b** Immunofluorescence analysis of primary antibodies and their isotype-specific immunoglobulins (IgG) in OCC (**a**) and the oviductal ampulla (**b**) isolated from mice after hCG injection (at 13 h post-hCG treatment). The isotype-specific IgG served as a negative control. Nuclei were counterstained by DAPI (blue). Scale bars represent 50 μm. The image is the representative of three independent experiments.

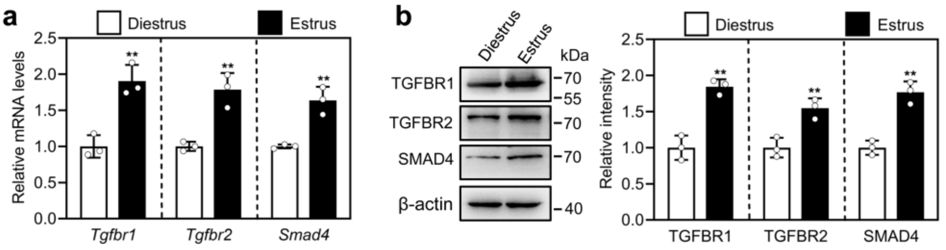

Supplementary Fig. 4 Comparison of TGFBR and SMAD4 gene and protein levels in diestrous and estrous mice. **a**, **b** The mRNA (**a**) and protein (**b**) levels of TGFBR1, TGFBR2, and SMAD4 in the oviductal ampullae isolated from diestrous and estrous mice (n = 3 independent experiments). The blot is the representative of three independent experiments. β-actin was used as a loading control. Bars indicate the mean ± SD. Each data point represents a biologically independent experiment. Statistical analysis was performed by two-tailed unpaired Student's t-test. $^{**}P < 0.01$.

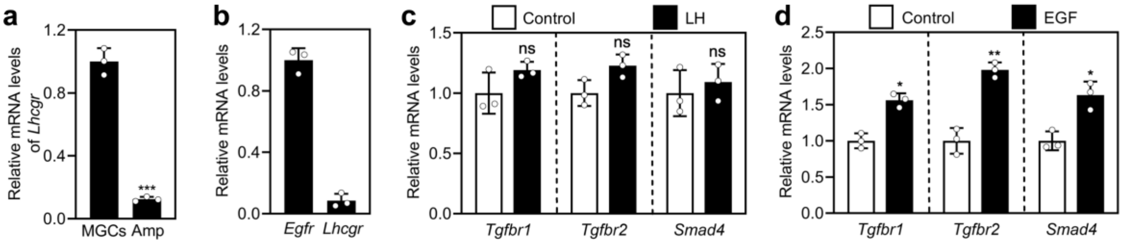

Supplementary Fig. 5 Effects of LH and EGF on the expression of *Tgfbr1*, *Tgfbr2*, and *Smad4* mRNA in the cultured ampullae. **a** Comparison of steady-state levels of *Lhcgr* mRNA in mural granulosa cells (MGCs) and oviductal ampullae (Amp). MGCs and ampullae were isolated from mice before and after hCG injection (at 13 h post-hCG treatment), respectively ($n = 3$ independent experiments). **b** The mRNA levels of *Egfr* and *Lhcgr* in oviductal ampullae isolated from mice after hCG injection (at 13 h post-hCG treatment). ($n = 3$ independent experiments). **c**, **d** The effect of LH (**c**) and EGF (**d**) on the expression of *Tgfbr1*, *Tgfbr2*, and *Smad4* mRNA ($n = 3$ independent experiments). Ampullae isolated from eCG-primed mice were cultured in medium supplemented with LH (1 μg/ml) or EGF (10 ng/ml) for 12 h. Bars indicate the mean ± SD. Each data point represents a biologically independent experiment. Statistical analysis was performed by two-tailed unpaired Student's *t*-test. ns, no significance ($P \geq 0.05$). *$P < 0.05$, **$P < 0.01$, and ***$P < 0.001$.

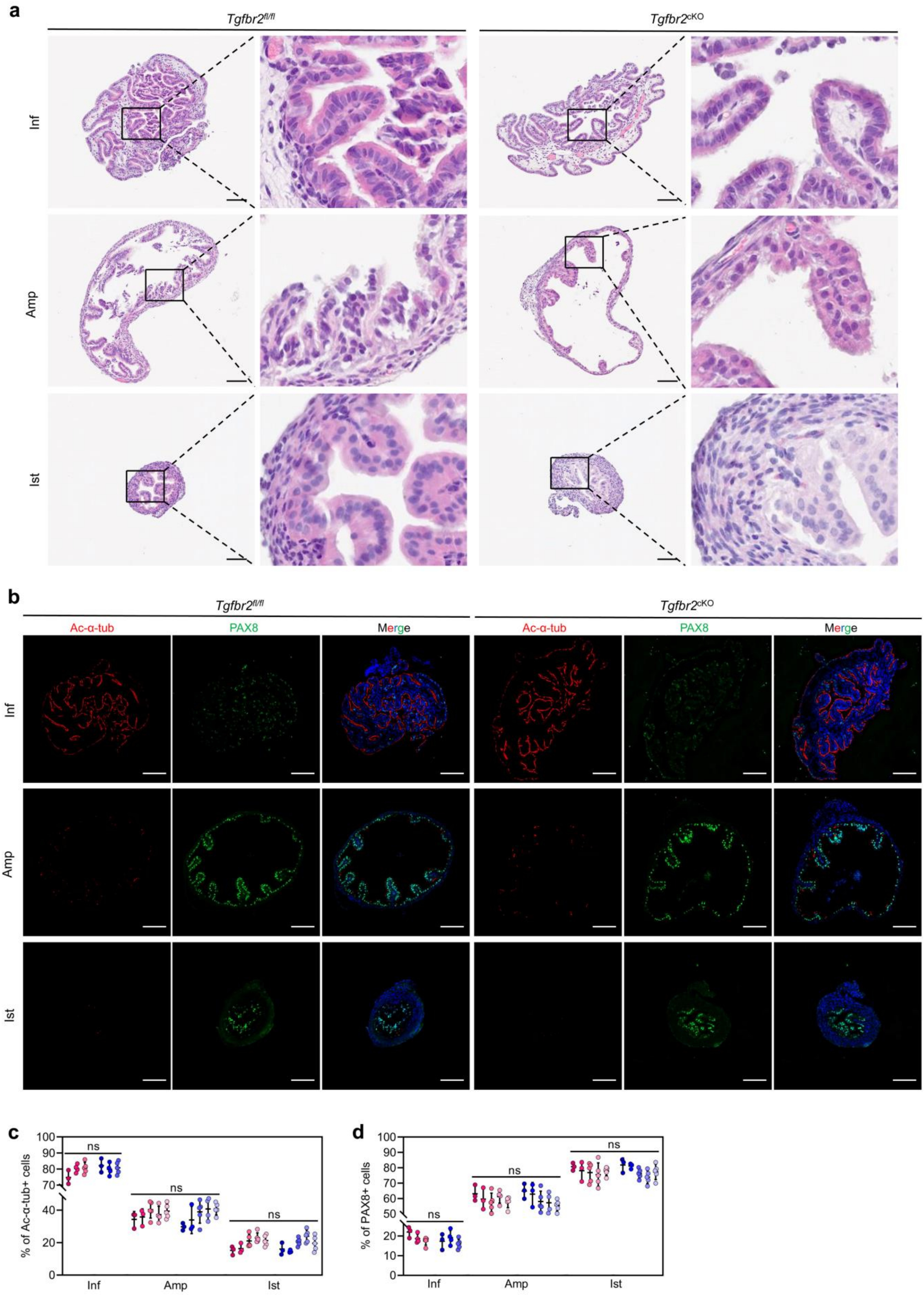

a
Tgfbr2fl/fl
Tgfbr2cKO
Inf
Amp
Ist
b
Ac-α-tub
PAX8
Merge
c
% of Ac-α-tub+ cells
ns
Inf
Amp
Ist
d
% of PAX8+ cells

85

Supplementary Fig. 6 Histological analysis of oviduct and the distribution and the relative number of ciliated and secretory cells in the oviducts of *Tgfbr2*$^{fl/fl}$ and *Tgfbr2*^{cKO} mice. **a** Histological analysis of oviductal infundibulum, ampulla, and isthmus from *Tgfbr2*$^{fl/fl}$ and *Tgfbr2*^{cKO} superovulated mice at 13 h post-hCG treatment. The amplified views of the boxed area are shown on the right-hand side. **b** Immunofluorescence analysis of acetylated-α-tubulin (a ciliated cell marker, red) and PAX8 (a secretory cell marker, green) in the oviductal infundibulum, ampulla, and isthmus of *Tgfbr2*$^{fl/fl}$ and *Tgfbr2*^{cKO} mice. Nuclei were counterstained by DAPI (blue). The image is the representative example of at least three independent experiments. **c**, **d** The percentage of epithelial cell with acetylated-α-tubulin- (**c**) and PAX8-positive signals (**d**) in oviductal infundibulum, ampulla, and isthmus (n = 3-5 mice in each group). The number of ciliated and secretory cells positively stained for acetylated-α-tubulin and PAX8, respectively, were counted in a minimum of three sections per each oviduct. One oviduct of each animal was randomly selected. Each shade of colour represents a oviduct, *Tgfbr2*$^{fl/fl}$ oviducts are in shades of red and *Tgfbr2*^{cKO} oviducts are in shades of blue. Scale bars represent 100 μm. Each data point represents a section. Statistical analysis was performed by two-tailed unpaired Student's *t*-test. ns, no significance ($P \geq 0.05$). Inf, infundibulum; Amp, ampulla; Ist, isthmus. Ac-α-tub, acetylated-α-tubulin.

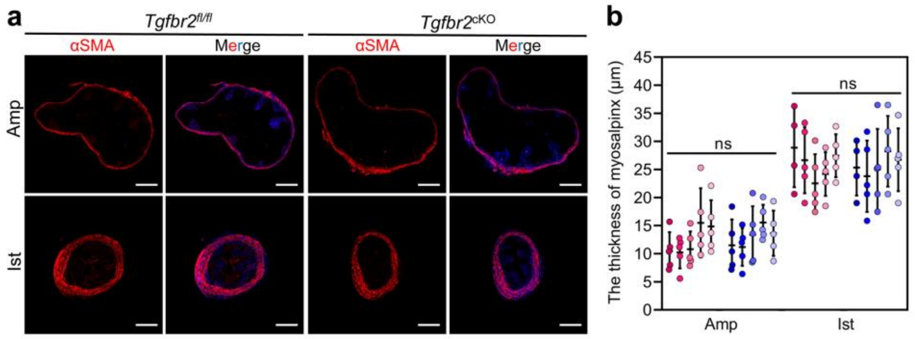

Supplementary Fig. 7 Conditional deletion of *Tgfbr2* in epithelial cells has no overt effects on oviductal smooth muscle. **a** Immunofluorescence analysis of smooth muscle α actin (αSMA, red) in the oviductal ampulla and isthmus of *Tgfbr2*$^{fl/fl}$ and *Tgfbr2*^{cKO} mice. Nuclei were counterstained by DAPI (blue). The image is the representative example of at least three independent experiments. Scale bars represent 100 μm. **b** The thickness of the myosalpinx in ampullary and isthmic regions of *Tgfbr2*$^{fl/fl}$ and *Tgfbr2*^{cKO} mice (n = 5 mice in each group). One oviduct of each animal was randomly selected and a minimum of three sections per oviduct were analyzed. Each shade of colour represents a oviduct, *Tgfbr2*$^{fl/fl}$ oviducts are in shades of red and *Tgfbr2*^{cKO} oviducts are in shades of blue. Each data point represents a section. Statistical analysis was performed by two-tailed unpaired Student's t-test. ns, no significance ($P \geq 0.05$). Amp, ampulla; Ist, isthmus.

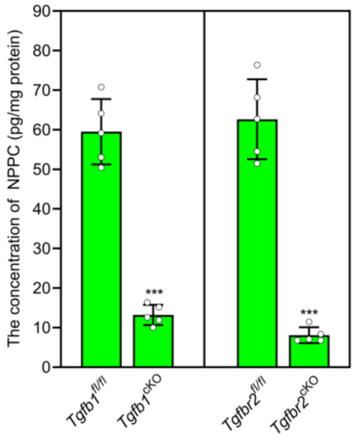

Supplementary Fig. 8 The NPPC levels in the ampullae of superovulated mice. The ampullae were collected from superovulated *Tgfb1*^{cKO}, *Tgfbr2*^{cKO}, and corresponding control mice at 13 h post-hCG treatment (n = 5 independent experiments). Each data point represents a biologically independent experiment. Bars indicate the mean ± SD. Statistical analysis was performed by two-tailed unpaired Student's t-test. ***$P < 0.001$.

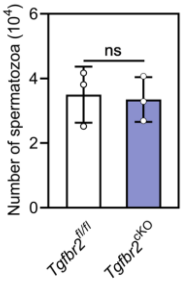

Supplementary Fig. 9 The total number of spermatozoa in the oviduct. The oviducts were isolated from *Tgfbr2*$^{fl/fl}$ and *Tgfbr2*^{cKO} mice at ~3 h post-copulation, and the flushed-out spermatozoa were counted under light microscope (n = 3 mice in each group). Each data point represents a single mouse. Bars indicate the mean ± SD. Statistical analysis was performed by two-tailed unpaired Student's *t*-test. ns, no significance.

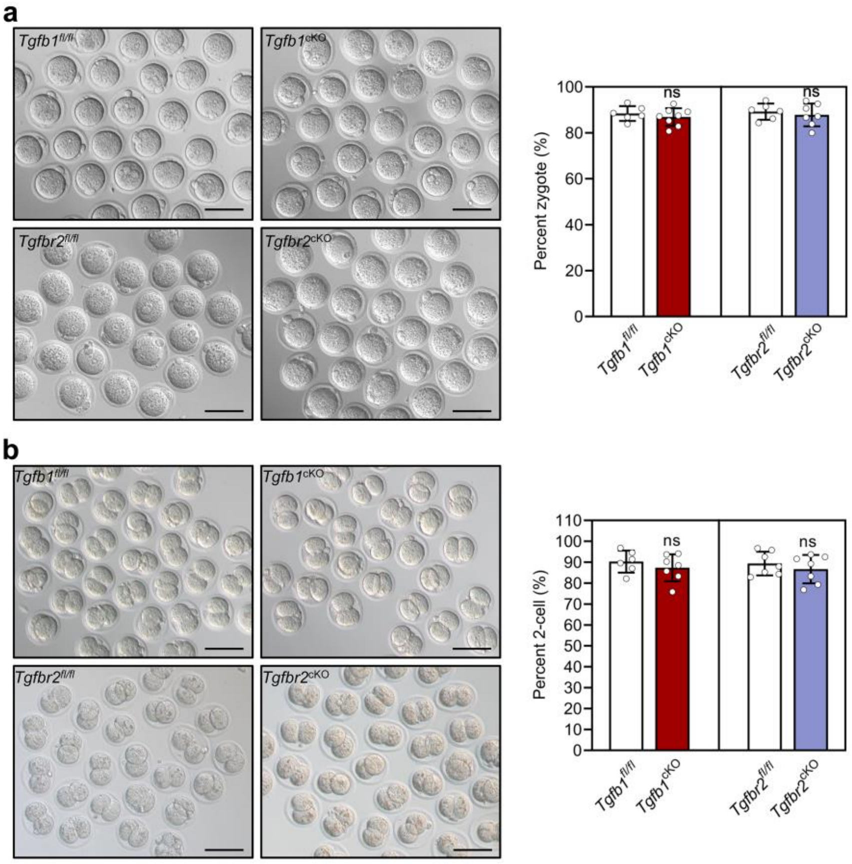

Supplementary Fig. 10 The effects of *Tgfb1* deletion in cumulus cells and *Tgfbr2* deletion in oviductal epithelial cells on the *in vitro* fertilization. **a** Representative image and rate of zygotes formed by ovulated oocytes after IVF. Oocytes were obtained from superovulated *Tgfb1*^{cKO}, *Tgfbr2*^{cKO}, and corresponding control mice at 13 h post-hCG treatment (n = 6-8 independent experiments). The fertilization rates were determined by visualizing the formation of pronuclei under a stereoscope at 8-9 h after IVF. **b** Representative image and rate of two-cell embryos formed by ovulated oocytes after IVF (n = 6-7 independent experiments). The image is the representative example of at least three independent experiments. Scale bars represent 100 μm. Bars indicate the mean ± SD. Each data point represents a biologically independent experiment. Statistical analysis was performed by two-tailed unpaired Student's t-test. ns, no significance ($P \geq 0.05$).

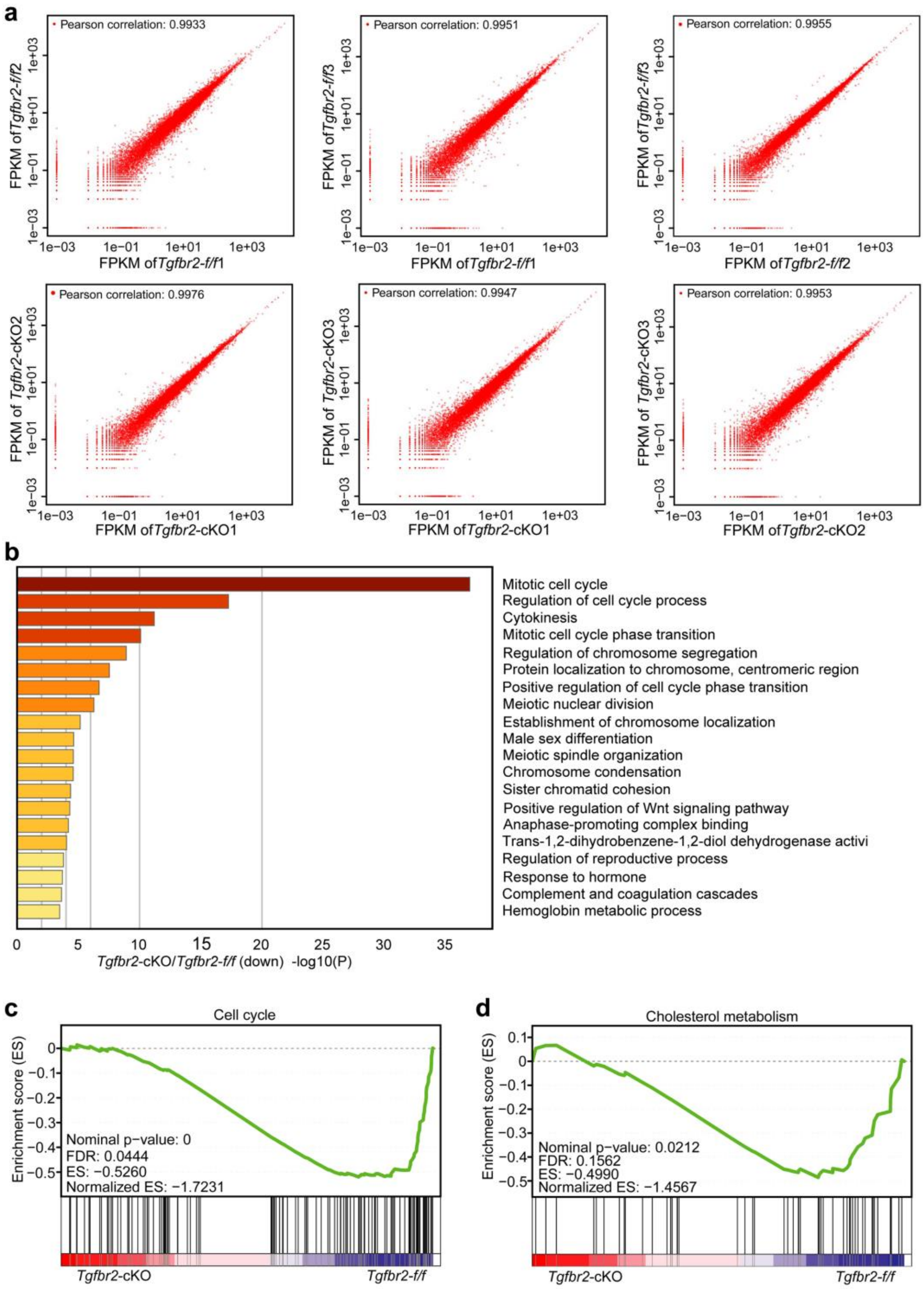

Supplementary Fig. 11 The oviduct transcriptome is distorted in *Tgfbr2*$^{\mathbf{cKO}}$ mice. **a** Scatterplot illustrating the pairwise scatterplots of the FPKM scores among two sample groups. **b** Bar graph showing the enriched GO/KEGG terms associated with the significantly downregulated transcripts in oviductal cells of *Tgfbr2*^{cKO} mice identified by RNA-seq, with the color indicating *P* value. (*n* = 3 mice in each group). **c**, **d** GSEA plots illustrating enrichment of gene sets of cell cycle (**c**) and cholesterol metabolism (**d**) in oviductal cells of *Tgfbr2*$^{fl/fl}$ and *Tgfbr2*^{cKO} mice (*n* = 3 mice in each group).

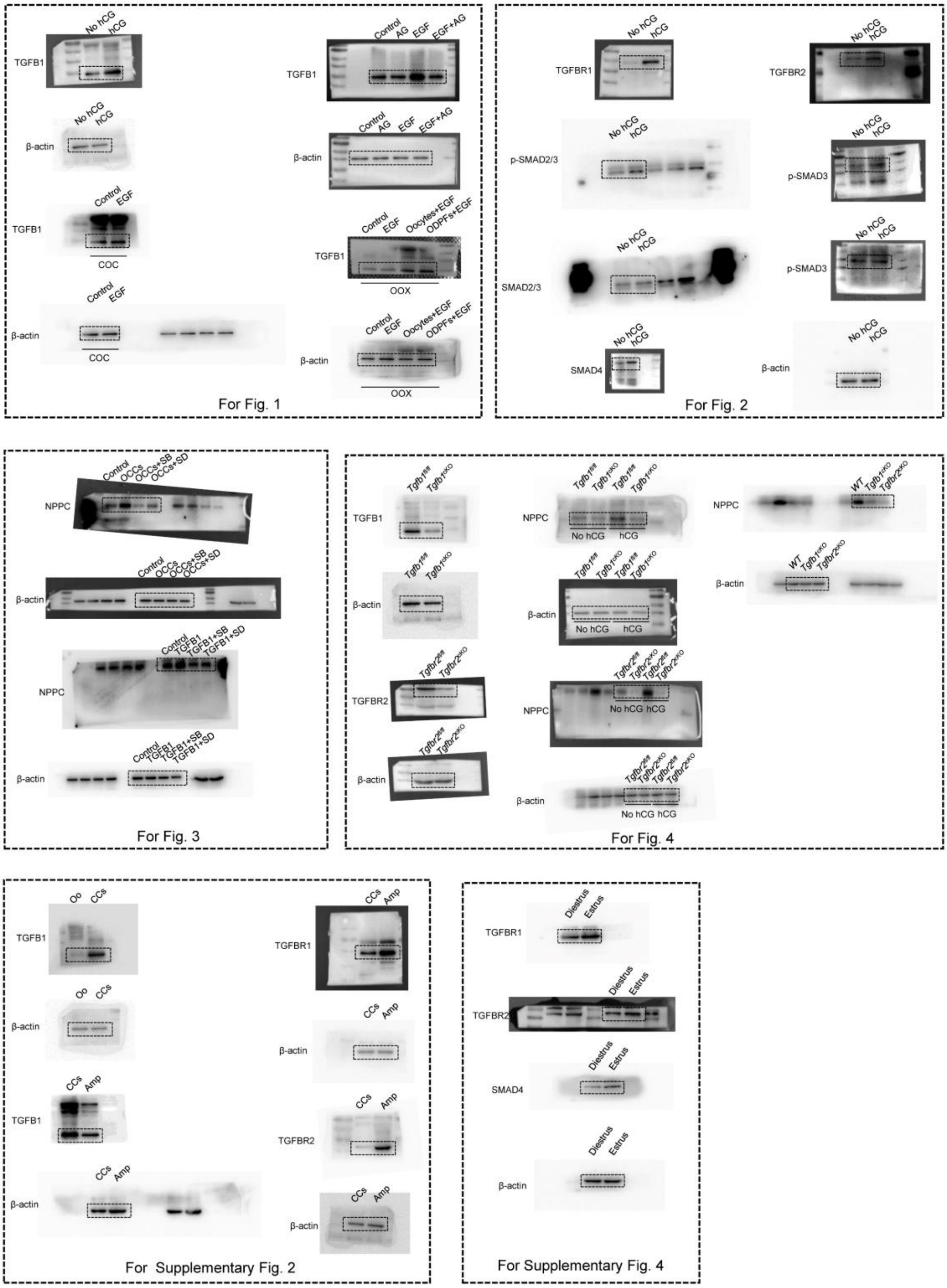

Supplementary Fig. 12 Uncropped scans of Western blotting results.

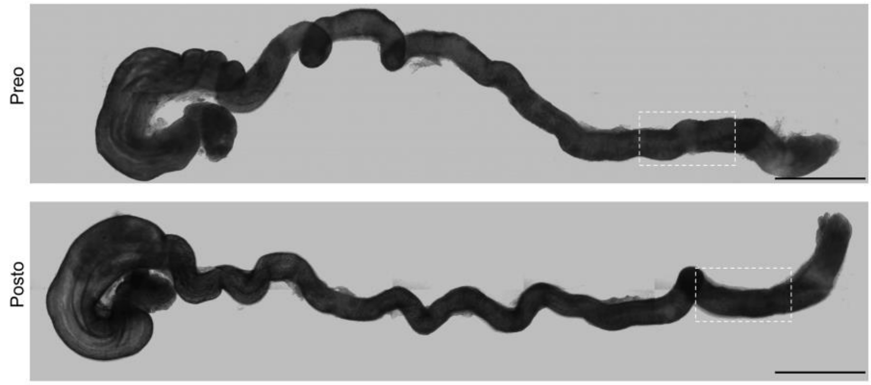

Supplementary Fig. 13 Site of the movie in the oviducts from preovulatory and postovulatory mice. Preovulatory and postovulatory oviducts were isolated from mice at a stated time, and were captured under a Zeiss LSM 880 confocal microscope by stitching. Dashed line box indicates the imaging region of the lower isthmus. Scale bars represent 1,000 μm.

Tables

Table 1. List of primary antibodies used in immune detection.

Antibody	Catalog Code	Source	Host	Dilution	
				IF	WB
acetylated-α-tubulin	T6793	Sigma	Mouse	1:500	
αSMA	ab124964	Abcam	Rabbit	1:500	
NPPC	sc374043	Santa Cruz Biotechnology	Rabbit		1:1000
PAX8	ab191870	Abcam	Rabbit	1:400	
p-SMAD2/3	8828	Cell Signaling Technology	Rabbit	1:100	1:1000
p-SMAD3	9520	Cell Signaling Technology	Rabbit	1:100	1:1000
SMAD2/3	8685	Cell Signaling Technology	Rabbit		1:1000
SMAD3	9523	Cell Signaling Technology	Rabbit		1:1000
SMAD4	46535	Cell Signaling Technology	Rabbit	1:100	1:1000
TGFB1	ab92486	Abcam	Rabbit	1:200	1:1000
TGFBR1	ab31013	Abcam	Rabbit	1:200	1:1000
TGFBR2	ab186838	Abcam	Rabbit	1:200	1:1000
β-actin	4967	Cell Signaling Technology	Rabbit		1:1000

IF, Immunofluorescence; WB, Western blotting

Table 2. Primer sequences for qRT-PCR.

Genes	Forward primer (5’-3’)	Reverse primer (5’-3’)
Agr3	CACTGATGGTTATTCACCACCTG	CCTGGGGTTCATACGTGTATAGT
Atp6v1c2	TCTGAGTTTTGGCTTATTTCGGC	TGTTGTGGGACAGGTTAGACTTT
Egfr	GCATCATGGGAGAGAACAACA	CTGCCATTGAACGTACCCAGA
Has1	TCTGTTACTGCCTCAATAAACCC	AAAGACTGCCCGGCTAGGT
Jam2	GTGTGGTGGAGCTACGATGC	TTTGGATTCCCTAGCAAACTTGT
Lama3	AAATGCTCCCTTCCCAAACT	ATCTCTCCCGTTGTTGATGG
Lhcgr	CGCCCGACTATCTCTCACCTA	GACAGATTGAGGAGGTTGTCAAA
Nppc	GGTCTGGGATGTTAGTGCAGCTA	TAAAAGCCACATTGCGTTGGA
Pdk4	CCGCTTAGTGAACACTCCTTC	TGACCAGCGTGTCTACAAACT
Press35	CCCTCTCTGATGGGTCGGAAA	TTCCTGACATTCGATGCCACA
Racgap1	TGCCCGTAATCAAGTGGACG	CTTGGCCTCGGTTGAGGAAAG
Smad2	ATGTCGTCCATCTTGCCATTC	AACCGTCCTGTTTTCTTTAGCTT
Smad3	CACGCAGAACGTGAACACC	GGCAGTAGATAACGTGAGGGA
Smad4	ACACCAACAAGTAACGATGCC	GCAAAGGTTTCACTTTCCCCA
Tgfb1	CTCCCGTGGCTTCTAGTGC	GCCTTAGTTTGGACAGGATCTG
Tgfb2	CTTCGACGTGACAGACGCT	GCAGGGGCAGTGTAAACTTATT
Tgfb3	CCTGGCCCTGCTGAACTTG	TTGATGTGGCCGAAGTCCAAC
Tgfbr1	TCTGCATTGCACTTATGCTGA	AAAGGGCGATCTAGTGATGGA
Tgfbr2	TTGGATTGCCAGTGCTAACCC	AACAAGCCACAGTAACATGACA
Rpl19	CTGAAGGTCAAAGGGAATGTGTTC	TGGTCAGCCAGGAGCTTCTTG
