# Supplementary material for: The oocyte cumulus complex regulates mouse sperm migration in the oviduct
Source: Commun Biol. 2022 Dec 3;5:1327. doi: 10.1038/s42003-022-04287-8 (PMC9719508; doi:10.1038/s42003-022-04287-8)
Supplement: Supplementary file 2 — Description of Additional Supplementary Files [file 42003_2022_4287_MOESM2_ESM.docx]

**Description of Additional Supplementary Files**

File name: Supplementary Audio 1

Description: NPPC promotes spermatozoa migration in preovulatory oviducts. Movement of spermatozoa in the lower isthmus of the oviducts isolated from preovulatory and postovulatory mice was observed by time-lapse imaging, related to Fig. 3c (left panel). Movies from the isthmic reservoirs of postovulatory oviducts (left panel) and preovulatory oviducts without (middle panel) and with NPPC (right panel) are shown side-by-side and have been timematched to the start. Scale bars represent 100 μm.

File name: Supplementary Audio 2

Description: Conditional deletion of Tgfb1 in cumulus cells blocks spermatozoa moving out of the isthmic reservoir. Movies from the isthmic reservoirs of postovulatory oviducts in Tgfb1fl/fl mice (left panel) and in Tgfb1cKO mice without (middle panel) and with NPPC (right panel) are shown side-by-side and have been time-matched to the start. Scale bars represent 100 μm.

File name: Supplementary Audio 3

Description: Conditional deletion of Tgfbr2 in oviductal epithelial cells blocks spermatozoa moving out of the isthmic reservoir. Movies from the isthmic reservoirs of postovulatory oviducts in Tgfbr2fl/fl mice (left panel) and in Tgfbr2cKO mice without (middle panel) and with NPPC (right panel) are shown side-byside and have been time-matched to the start. Scale bars represent 100 μm.

File name: Supplementary Data 1

Description: The significantly dysregulated transcripts in the oviductal cells of Tgfbr2cKO mice.

File name: Supplementary Data 2

Description: The source data underlying the graphs in the main figure.
