# Supplementary material for: The oocyte cumulus complex regulates mouse sperm migration in the oviduct
Source: Commun Biol. 2022 Dec 3;5:1327. doi: 10.1038/s42003-022-04287-8 (PMC9719508; doi:10.1038/s42003-022-04287-8)
Supplement: Supplementary file 8 — Reporting summary [file 42003_2022_4287_MOESM8_ESM.pdf]

Corresponding author(s): COMMSBIO-22-2024A
Meijia Zhang

Last updated by author(s): November 11, 2022

Reporting Summary

Nature Portfolio wishes to improve the reproducibility of the work that we publish. This form provides structure for consistency and transparency in reporting. For further information on Nature Portfolio policies, see our Editorial Policies and the Editorial Policy Checklist.

Statistics

For all statistical analyses, confirm that the following items are present in the figure legend, table legend, main text, or Methods section.

n/a	Confirmed	
☐	☒	The exact sample size (*n*) for each experimental group/condition, given as a discrete number and unit of measurement
☐	☒	A statement on whether measurements were taken from distinct samples or whether the same sample was measured repeatedly
☐	☒	The statistical test(s) used AND whether they are one- or two-sided<br>*Only common tests should be described solely by name; describe more complex techniques in the Methods section.*
☒	☐	A description of all covariates tested
☐	☒	A description of any assumptions or corrections, such as tests of normality and adjustment for multiple comparisons
☐	☒	A full description of the statistical parameters including central tendency (e.g. means) or other basic estimates (e.g. regression coefficient) AND variation (e.g. standard deviation) or associated estimates of uncertainty (e.g. confidence intervals)
☒	☐	For null hypothesis testing, the test statistic (e.g. *F*, *t*, *r*) with confidence intervals, effect sizes, degrees of freedom and *P* value noted<br>*Give P values as exact values whenever suitable.*
☒	☐	For Bayesian analysis, information on the choice of priors and Markov chain Monte Carlo settings
☒	☐	For hierarchical and complex designs, identification of the appropriate level for tests and full reporting of outcomes
☒	☐	Estimates of effect sizes (e.g. Cohen's *d*, Pearson's *r*), indicating how they were calculated

Our web collection on statistics for biologists contains articles on many of the points above.

Software and code

Policy information about availability of computer code

Data collection	Excel
Data analysis	Graphpad prism version 8.3

For manuscripts utilizing custom algorithms or software that are central to the research but not yet described in published literature, software must be made available to editors and reviewers. We strongly encourage code deposition in a community repository (e.g. GitHub). See the Nature Portfolio guidelines for submitting code & software for further information.

Data

Policy information about availability of data

All manuscripts must include a data availability statement. This statement should provide the following information, where applicable:
- Accession codes, unique identifiers, or web links for publicly available datasets
- A description of any restrictions on data availability
- For clinical datasets or third party data, please ensure that the statement adheres to our policy

All data needed to evaluate the conclusions in this study are present in the manuscript and/or Supplementary information, and available from the corresponding author on reasonable request. The RNA-seq data are deposited in the NCBI Sequence Read Archive (accession number PRJNA888862).

Human research participants

Policy information about studies involving human research participants and Sex and Gender in Research.

Reporting on sex and gender	This information has not been collected. No human sample are used in our study.
Population characteristics	*Describe the covariate-relevant population characteristics of the human research participants (e.g. age, genotypic information, past and current diagnosis and treatment categories). If you filled out the behavioural & social sciences study design questions and have nothing to add here, write "See above."*
Recruitment	*Describe how participants were recruited. Outline any potential self-selection bias or other biases that may be present and how these are likely to impact results.*
Ethics oversight	*Identify the organization(s) that approved the study protocol.*

Note that full information on the approval of the study protocol must also be provided in the manuscript.

Field-specific reporting

Please select the one below that is the best fit for your research. If you are not sure, read the appropriate sections before making your selection.

☒ Life sciences ☐ Behavioural & social sciences ☐ Ecological, evolutionary & environmental sciences

For a reference copy of the document with all sections, see nature.com/documents/nr-reporting-summary-flat.pdf

Life sciences study design

All studies must disclose on these points even when the disclosure is negative.

Sample size	There are no statistical methods to predetermine sample size in this study. In vivo experiments were conducted in adherence to the 3R principle, and numbers in each group were determined based on the published studies of similar programs. In vitro experiments, numbers in each group were determined as the minimum number of mice were required to achieve statistical significance between the treatment groups and the corresponding control groups.
Data exclusions	No data were excluded.
Replication	For western blotting, immunofluorescence, histology, qRT-PCR, NPPC measurement, and RNA-seq analyses, the experiments were repeated for 3-5 times. For spermatoza migration, ovulation, fertilization, and fertility analyses, the experiments were repeated for 5-17 times.
Randomization	For the animal study, the mice in the treatment/knockout groups and the corresponding control groups delivered within the same week and derived from the same breeding cage.
Blinding	Investigators were blinded in where possible.

Reporting for specific materials, systems and methods

We require information from authors about some types of materials, experimental systems and methods used in many studies. Here, indicate whether each material, system or method listed is relevant to your study. If you are not sure if a list item applies to your research, read the appropriate section before selecting a response.

Materials & experimental systems

n/a	Involved in the study	
☐	☒	Antibodies
☒	☐	Eukaryotic cell lines
☒	☐	Palaeontology and archaeology
☐	☒	Animals and other organisms
☒	☐	Clinical data
☒	☐	Dual use research of concern

Methods

n/a	Involved in the study	
☒	☐	ChIP-seq
☒	☐	Flow cytometry
☒	☐	MRI-based neuroimaging

Antibodies

Antibodies used	The following antibodies were used for western blotting and immunofluorescence: mouse anti-acetylated-α-tubulin (Sigma, T6793), rabbit anti-αSMA (Abcam, ab124964), rabbit anti-NPPC (Santa Cruz Biotechnology, sc374043), rabbit anti-PAX8 (Abcam, ab191870), rabbit anti-p-SMAD2/3 (Cell Signaling Technology, 8828), rabbit anti-p-SMAD3 (Cell Signaling Technology, 9520), rabbit anti-SMAD2/3 (Cell Signaling Technology, 8685), rabbit anti-SMAD3 (Cell Signaling Technology, 9523), rabbit anti-SMAD4 (Cell Signaling Technology, 46535), rabbit anti-TGFB1 (Abcam, ab92486), rabbit anti-TGFBR1 (Abcam, ab31013), rabbit anti-TGFBR2 (Abcam, ab186838), rabbit anti- β-actin (Cell Signaling Technology, 4967). The detailed information for the antibodies was presented in the supplementary information.
Validation	All antibodies used in this study are purchased from companies and validated in numberous published papers or in manufacturer's website. For immunofluorescence, We have set the controls of TGFB1, TGFBR1, TGFBR2, p-SMAD23, p-SMAD3, and SMAD4 using isotype-specific immunoglobulins at the same protein concentration as the primary antibodies.

Animals and other research organisms

Policy information about studies involving animals; ARRIVE guidelines recommended for reporting animal research, and Sex and Gender in Research

Laboratory animals	Tgfb1fl/fl, Tgfbr2fl/fl, and Wnt7a–Cre mice were purchased from The Jackson Laboratory (Bar Harbor, ME, USA). Mice with Tgfb1 deletions in the cumulus cells were generated by crossing Tgfb1fl/fl mice with Fshr–Cre mice. Mice with Tgfbr2 deletions in the epithelial cells were generated by crossing Tgfbr2fl/fl mice with Wnt7a–Cre mice. Fshr–Cre mice were obtained from Dr. L. Dubeau (University of Southern California). B6D2-Tg (CAG/su9-DsRed2, Acr3-EGFP) RBGS002Osb (RBGS) male mice using for monitoring spermatozoa movement were purchased from RIKEN BioResource Center (Ibaraki, Tsukuba, Japan). Female mice (21-23 days or six-week-old) and male mice (10-week-old) were used in this study.
Wild animals	Female C57BL/6J wild mice are used in this study.
Reporting on sex	The female and male mice were used in this study.
Field-collected samples	This study did not involve field-collected samples.
Ethics oversight	Mouse experimental procedures and protocols were approved by the Institutional Animal Care and Use Committee of South China University of Technology and were conducted in accordance with the institutional guides for the care and use of laboratory animals.

Note that full information on the approval of the study protocol must also be provided in the manuscript.
